# Unbiased multitissue transcriptomic analysis reveals complex neuroendocrine regulatory networks mediated by spinal cord injury-induced immunodeficiency

**DOI:** 10.1186/s12974-023-02906-7

**Published:** 2023-09-30

**Authors:** Hong Zeng, Li Cheng, De-zhi Lu, Shuai Fan, Ke-xin Wang, Li-li Xu, Bin Cai, Mou-wang Zhou, Jin-wu Wang

**Affiliations:** 1 Department of Rehabilitation Medicine, Shanghai Ninth People’s Hospital Affiliated to Shanghai Jiao Tong University School of Medicine, 500 Quxi Road, Shanghai, 200011 China; 2 Department of Orthopedic Surgery, Shanghai Ninth People’s Hospital Affiliated to Shanghai Jiao Tong University School of Medicine, 639 Zhizaoju Road, Shanghai, 200011 China; 3 Department of Rehabilitation Medicine, Peking University Third Hospital, 49 North Garden Road, Beijing, 100191 China; 4 School of Medicine, Shanghai University, Shanghai, 200444 China

**Keywords:** SCI-induced immunodeficiency syndrome, Transcriptome, Neuroendocrine immunomodulatory axis, Neuroinflammation, Hypothalamo-pituitary-adrenal axis

## Abstract

**Background:**

Spinal cord injury (SCI), which causes loss of sensory and motor function in the body below the level of injury, is a devastating disease of the central nervous system. SCI leads to severe secondary immunosuppression, called SCI-induced immunodeficiency syndrome (SCI-IDS), which is characterized by increased susceptibility to infection and further exacerbates neurological dysfunction. Several studies have suggested that SCI-IDS is an independent risk factor for poor neurological prognosis. SCI-IDS predominantly occurs following injury above the T5 levels and eventually leads to systemic immune failure, possibly via the sympathetic–adrenal medullary axis and the hypothalamic‒pituitary‒adrenal (HPA) axis. However, the mechanism remains unclear.

**Methods and objectives:**

The concentrations of adrenocorticotropic hormone and cortisol in plasma, as well as changes in sympathetic activity (blood pressure and catecholamine levels in plasma), were assessed in rats in the high-level (T3) spinal cord injury (T3-SCI) group and the low-level (T10) spinal cord injury (T10-SCI) group. Second, the differential regulation of the gene network between the sympathetic–adrenal medullary axis and the HPA axis was explored by histology and multitissue transcriptomics, and the neuroendocrine–immune network associated with SCI-IDS was further elucidated.

**Results:**

The spleen and thymus gland, which are secondary immune organs, were significantly atrophied in rats in the T3-SCI group, and the white pulp of the spleen was significantly atrophied. The level of cortisol, which is mediated by the adrenal glands, was markedly elevated, but norepinephrine levels were markedly decreased. There was no difference in adrenocorticotropic hormone expression between any of the groups. The transcriptome analysis results showed that the downregulated differentially expressed genes (DEGs) in the T3-SCI group were enriched in the GO term immunoregulation, indicating that splenic immune function was markedly impaired after high-level SCI. The upregulated DEGs in the hypothalamus (hub genes: *Nod2, Serpine1, Cebpb, Nfkbil1, Ripk2, Zfp36, Traf6, Akap8, Gfer, Cxcl10, Tnfaip3, Icam1, Fcgr2b, Ager, Dusp10,* and *Mapkapk2*) were significantly enriched in inflammatory pathways, and the downregulated genes (hub genes: *Grm4, Nmu, P2ry12, rt1-bb1, Oprm1, Zfhx2, Gpr83,* and *Chrm2*) were enriched in pathways related to inhibitory Gi-mediated G protein-coupled receptor (Gi-GPCR) neurons and neuropeptide changes. The upregulated genes in the adrenal glands (hub genes: *Ciart, per2, per3, cry1,* and *cry2*) were enriched in cortisol secretion and circadian rhythm changes, and the downregulated genes (hub genes: *IL7r, rt1-bb, rt1-bb1, rt1-da, rt1-ba, cd74, cxcr3, vcam1, ccl5, bin1,* and *IL8*) were significantly enriched in MHC-mediated immune responses.

**Conclusions:**

To explore the possible mechanism underlying SCI-IDS, this study assessed the differential regulation of the gene network associated with neuroendocrine immunity after SCI. Progressive neuroinflammation spreads after injury, and neurotransmission through Gi-mediated G protein-coupled receptors in the HPA axis and neuropeptide production by the hypothalamus are inhibited. Disruption of the connection between the hypothalamus and the adrenal glands causes autonomous regulation of the adrenal glands, disturbance of circadian rhythm and finally hypercortisolemia, leading to general suppression of peripheral adaptive immunity. Neuraxial nerve inflammation caused by SCI persists indefinitely, blocking nerve repair; persistent system-wide immunosuppression in the periphery results in increased susceptibility to infection, leading to poor neurological prognosis.

**Supplementary Information:**

The online version contains supplementary material available at 10.1186/s12974-023-02906-7.

## Introduction

Spinal cord injury (SCI) is a devastating disease of the central nervous system (CNS) that causes loss of sensation below the level of injury, resulting in motor and autonomic dysfunction [1, 2]. SCI can also impair immune function and increase the risk of infection, resulting in SCI-related pneumonia, urinary and gastrointestinal infections, bedsores and wound infections, and even osteomyelitis [3]. In fact, infection is common post-SCI, and more than 50% of deaths from SCI are caused by infection, mainly due to a sharp decline in the number of immune cells and significant dysfunction of these cells after injury [4]. The clinicopathological manifestations of SCI-induced immunosuppression include circulating lymphopenia, impaired lymphocyte function, and atrophy of immune organs such as the spleen, thymus, and secondary lymphoid organs. This severe secondary immunosuppression caused by SCI is called SCI-induced immunodeficiency syndrome (SCI-IDS) [3–7], which is characterized by increased susceptibility to infection, which further exacerbates neurological deficits. Several studies have suggested that SCI-IDS is also an independent risk factor for poor neurological prognosis [6]. SCI has been shown to result in permanent impairment of the neurogenic immune response, leading to chronic immune dysfunction [7]. The mechanisms underlying this immune dysfunction remain unclear, and effective treatments and interventions are lacking. Infections due to immunodeficiency remain the most common obstacle to survival and recovery in people with SCI.

Complete SCI results in severe immunodeficiency. Whether SCI-IDS occurs is dependent on the level of injury, and SCI-IDS is particularly common after high-level (T5-T6 and above) injury; however, the occurrence of SCI-IDS is not level-dependent in incomplete SCI [8]. At present, it is generally believed that the mechanism of SCI-IDS involves immune dysfunction caused by large-scale modification of neural networks in the spinal canal and spinal cord centre after injury [9], mainly due to impairment of neuroendocrine signalling and disruption of sympathetic neural networks [7]. Among these neural networks, alterations to three related neural axes, namely, the sympathetic–adrenal medullary axis (SAM), the HPA axis, and the vagus–cholinergic axis, which have anti-inflammatory effects, may eventually lead to systemic immune failure [6, 7, 10].

The hypothalamus is the central integration region of the SAM and HPA axes, and this region is closely connected with circumventricular organs (CVOs), other brain structures (e.g., the hippocampus), peripheral autonomic nerves, and endocrine-related brain regions that lack a blood‒brain barrier (BBB) [11, 12]. The hypothalamus recognizes and senses the spread of CNS neuroinflammation and maintains homeostasis by regulating hormone secretion and feedback [11, 12]. The adrenal glands are important peripheral endocrine organs that play a substantial role in maintaining the body's homeostasis. The adrenal glands are made up of two distinct anatomical regions: the cortex, which produces steroid hormones, including glucocorticoids (GCs), and the medulla, which is innervated by sympathetic postganglionic neurons that synthesize and release catecholamines (CAs) [3, 13]. The levels of GCs and CAs change significantly after SCI, and although these stress hormones are thought to be associated with central nervous system injury-induced immunodeficiency syndrome (CIDS) [14], the neurological, endocrine, and immune changes caused by damage to peripheral organs and the central nervous system after injury remain unclear.

Therefore, in this study, we performed unbiased multitissue transcriptomics analysis to analyse the possible underlying mechanisms of immune deficiency after T3 SCI. Here, we analysed the differential regulation of neuroendocrine pathways associated with immunosuppression after high-level injury by comparing the transcriptomes of the hypothalamus, spinal cord, adrenal glands, and spleen in rats with high-level (T3) and low-level (T10) SCI. In this preliminary first-in-class study, we mapped and elucidated the gene network of the neuroendocrine–immune axis associated with SCI-IDS, providing new insights into SCI-mediated immune deficiency and a new direction for future studies on immunosuppression.

## Methods

### Animals and groupings

SPF-grade adult male Sprague‒Dawley (SD) rats (Shanghai SIPPR-BK Laboratory Animal Co., Ltd., Shanghai, China) were used in this study. We used male rats for several reasons: first, the incidence of male patients with traumatic spinal cord injury is significantly higher than that in women, so we selected male rat models [15, 16]; second, we investigated neuroendocrine changes after SCI, while hormonal fluctuations in the estrous cycle in female animals increased the variability of the findings; third, male animals did not have periodic physiological index fluctuations of male and female animals, and the hormone levels in the body were stable, which met the needs of this study [17]. All rats were housed in separate cages on a 12-h light–dark cycle at 23 ± 1 °C and 50% relative humidity, with ad libitum access to food and water. All rats were acclimated to the environment for at least 1 week prior to the experiment and maintained in accordance with the guidelines for the care and use of experimental animals. All procedures and euthanasia protocols performed in the study were reviewed and approved by the Animal Care and Use Committee (IACUC) of Shanghai Ninth People’s Hospital.

**Fig. 1:**
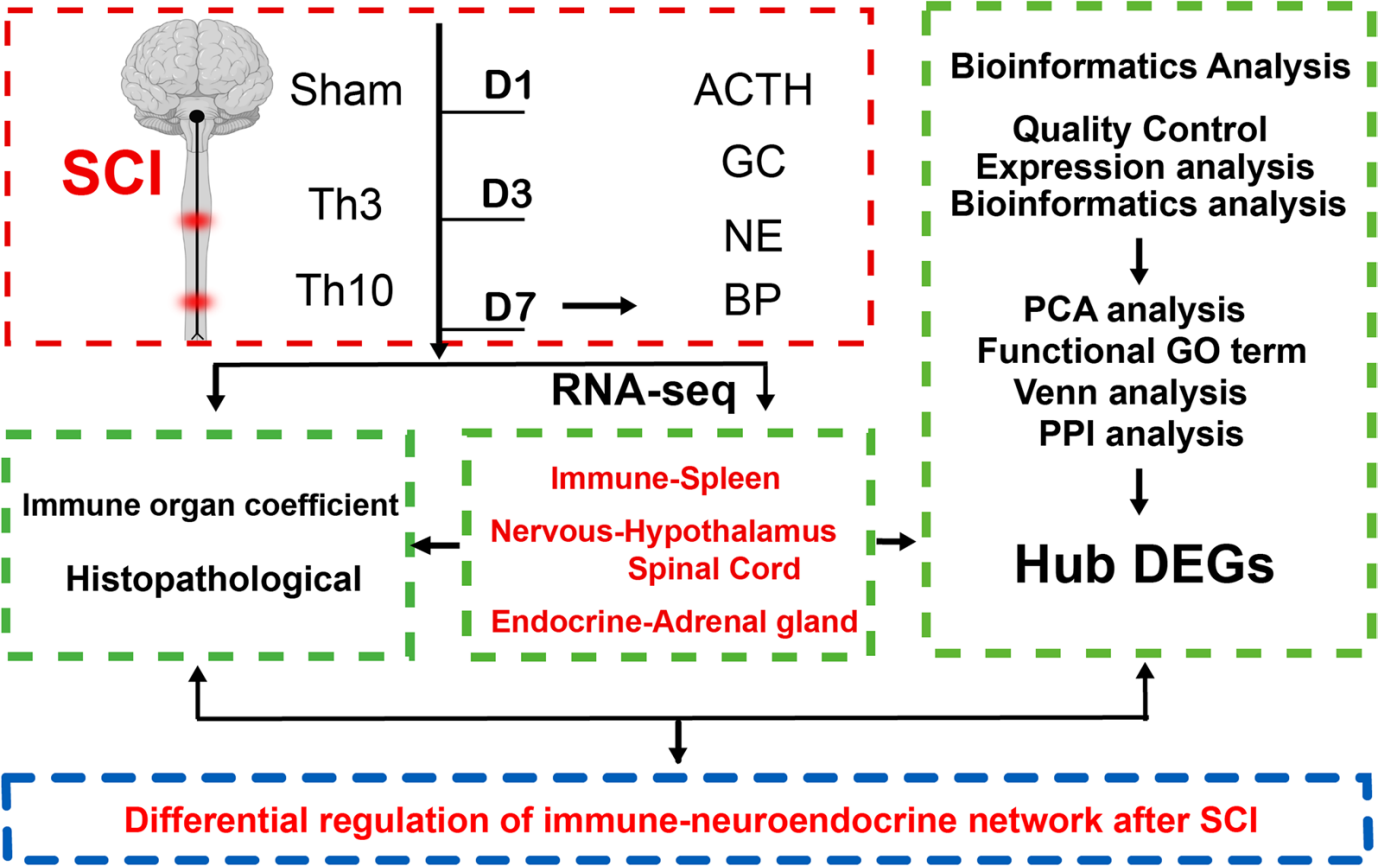
Schematic of the experimental design

The animals were randomly divided into the following three groups, with 40 animals in each group: (1) the sham surgery group (referred to as the sham group); (2) the T3 spinal cord transection group (referred to as the T3-SCI group); and (3) the T10 spinal cord transection group (referred to as the T10-SCI group). The entire experimental procedure is shown in Fig. 1.

### Establishment of a transection SCI model in rats

All rats received the antibiotic ampicillin sodium (80 mg/kg; Qingdao Xinxing Pharmaceutical Co., Ltd., China) as a prophylactic for three days. The animals were anaesthetized with a ketamine/xylazine mixture (50 mg/kg KetaVed (Henry Schein) and 5 mg/kg xylazine (AnaSed)) by intraperitoneal injection and given appropriate analgesics (5 mg/kg flunixin, subcutaneous). After anaesthesia, the animals were placed in the prone position on an operating table, the limbs were fixed, and the upper chest was raised using a cotton pad. The dorsal skin was incised from C8-T4 lateral to the T2 vertebra, the muscle tissue over the spinal cord was removed layer by layer, the thoracic T3 segment was located, and the lamina over the T3 segment was removed under an operating microscope, exposing the spinal cord. In the sham group, the incision was closed layer by layer after exposure of the spinal cord (without injury), while in the T3-SCI group, the spinal cord was transected with microscissors under a microscope after exposure. Rats in the T10-SCI group underwent complete spinal cord transection at the T10 level via the same procedure after exposure of the T10 spinal cord. Successful construction of the rat spinal cord injury models was confirmed by manifestations such as spastic convulsions of both lower extremities and spastic tail swing. After the bleeding had fully stopped, the dorsal tissues of the rats were sutured layer by layer, and the rats were allowed to recover on a heated blanket and placed in a clean cage for observation. Beginning immediately after surgery, the rats were injected subcutaneously with warm Ringer's sodium lactate solution (5 mL) and ampicillin sodium twice daily (morning and afternoon) until day 3 after injury. After surgery, the bladder was expressed 3 times a day to assist with urination until spontaneous urination resumed. All evaluations and analyses were performed by experienced investigators who were not aware of the experimental design.

### Monitoring of tail artery blood pressure in rats

Blood pressure was monitored in each group with a noninvasive rat tail artery measurement instrument (BP-2010A, Softron Biotechnology Co., Ltd., Beijing China) according to the manufacturer's protocol. The average of 3 consecutive measurements was calculated. Measurements were taken in an unmanned, quiet, and warm environment after the rats had calmed down. The systolic blood pressure (SBP) of the rats was measured on days 1, 3, and 7 postoperatively [18].

### Determination of immune organ indices

After the rats were euthanized, the thymus and spleen were weighed separately, and the thymus index and spleen index were calculated. The thymus index (mg/g) was calculated according to the following formula: thymus index (mg)/thymus weight (mg)/rat body weight (g). The spleen index (mg/g) was calculated as follows: spleen weight (g)/rat body weight (g) [19].

### Tissue preparation

At a predetermined time point, after anaesthesia, the rat diaphragm was cut, and the pericardium was opened. After blood collection from the left apex, 5 mm pieces of tissue from the hypothalamus, adrenal glands, spleen and T3 spinal cord were quickly collected from rats in each group, placed in cryopreservation tubes, frozen in liquid nitrogen, and stored in a low-temperature freezer at − 80 °C. Other rats were perfused with 150 mL of sterile 0.9% normal saline and 300 mL of 4% paraformaldehyde (Biosharp, Beijing, China) after blood collection, and the tissues mentioned above were placed in paraformaldehyde overnight. After dehydration in xylene and an alcohol gradient and embedding in paraffin, the samples were cut into 5 µm continuous sections with a microtome or incubated in a sucrose gradient (10%, 20%, and 30%), embedded in OCT compound, and cut into 20 µm continuous frozen sections with a microtome (Leica, Germany).

### Pathological staining of paraffin adrenal sections

On the 7th day, paraffin sections from each group were baked at 60 °C; soaked in xylene I and II for 30 min; soaked for 5 min each in 100%, 100%, 95%, 95% and 80% alcohol; and rinsed twice with double-distilled water.

1. HE staining
  The tissues were stained with hematoxylin for 1 min, rinsed in double-distilled water, differentiated in 1% hydrochloric acid, rinsed with double-distilled water, and incubated with eosin (H&E; Sigma‒Aldrich, St. Louis, MO, USA) for 2 min.
2. Immunohistochemistry
  For immunohistochemistry, 5 µm paraffin sections were dewaxed in water and incubated with sodium citrate buffer (10 mM, pH 6.0; Boster Biological Technology, Ltd. Wuhan China) in a pressure cooker for antigen retrieval. The tissues were then blocked with 3% H_2_O_2_ to quench endogenous peroxidase activity, blocked with 10% sheep serum (Boster Biological Technology, Ltd. Wuhan China) for 30 min, incubated with rabbit anti-tyrosine hydroxylase (TH, 1:100; Bioss, Beijing, China) primary antibody at 37 °C for 2 h, rinsed with PBS, incubated with anti-rabbit IgG secondary antibody (1:100; Bioss, Beijing, China) for 30 min, rinsed with water, incubated with DAB (Bioss, Beijing, China) for colour development, counterstained with haematoxylin, dehydrated in graded alcohol for 2 min each, placed in xylene I and II for 5 min each, and sealed with neutral resin. All staining and immunohistochemical staining images were captured under a NanoZoomer Digital Pathology microscope (Hamamatsu). There were 4 animals per group.

### Immunofluorescence

Frozen sections were thawed for 30 min at room temperature, washed three times (10 min each) in 0.1 mmol/L PBS (PBS-TX) containing 0.1% Triton X-100 (Sigma‒Aldrich, St. Louis, MO, USA) and placed in permeabilization blocking buffer (0.1 mmol/L PBS, pH 7.3, 0.5% Triton) at 37 °C for 15 min. The sections were blocked with 10% (v/v) goat serum (Bioss, Beijing, China) for another 30 min. The sections were then incubated with primary antibody overnight at 4 °C. After washing with PBS-TX the next day (3 times for 10 min each), the sections were incubated with the secondary antibody for 1 h at room temperature and then washed with PBS-TX. Nuclei were stained with 4,6-diamidino-2-phenylindole (DAPI, 1 μg/mL; Sigma‒Aldrich) for 5 min, and the slides were sealed with anti-fluorescent quenching agent after washing with PBS-TX. Images were captured under a Leica TCS STED confocal fluorescence microscope (Leica Microsystems Inc., Wetzlar, Germany). For the negative control, the corresponding allotypic serum was used instead of primary antibody.

The following antibodies were used at the indicated dilutions: rabbit anti-Iba1 (1:200; GeneTex, Inc., USA), chicken anti-GFAP (1:500; Abcam, Cambridge, MA, USA), and mouse anti-PSD-95 (1:200; Abcam, Cambridge, MA, USA). The fluorescent secondary antibodies included Alexa Fluor 594-conjugated AffiniPure goat anti-rabbit IgG (H + L) (1:800; Jackson ImmunoResearch Laboratories, West Grove, PA, USA); Alexa Fluor 685-conjugated AffiniPure goat anti-mouse IgG (H + L) (1:400; Jackson ImmunoResearch Laboratories, West Grove, PA, USA); and Alexa Fluor 488-conjugated AffiniPure goat anti-chicken IgG (H + L) (1:400; Jackson ImmunoResearch Laboratories, West Grove, PA, USA). Three slices each from 4 animals per group (all rats in the same group underwent injury at the same level) were used for fluorescence quantification; the number of positive cells or the mean optical density (mean optical density = integrated optical density (IOD)/area) was determined. The experimenters who performed the analysis were unaware of the group identities of the rats.

### Enzyme-linked immunosorbent assay (ELISA)

Serum samples were obtained by centrifugation after apical blood collection in anticoagulant blood collection vessels; blood sampling was performed at the same time of day to ensure standardization and reduce the impact of circadian rhythm on plasma protein levels. The concentrations of norepinephrine (NE, Demeditec Diagnostics GmbH, Germany), adrenocorticotropic hormone (ACTH, Abcam, Cambridge, MA, USA) and cortisol (GC, Coibo Bio, Shanghai, China) in the peripheral plasma of rats were analysed on the 7th day after surgery by ELISA, which was performed according to the instructions provided by the manufacturer. The absorbance was measured at 450 nm with a Multi-Mode Microplate Reader (Varioskan Flash, Thermo Scientific Inc., USA). The concentrations of NE, ACTH, and GC were calculated according to standard curves.

### RNA extraction, library preparation, and Illumina HiSeq X Ten sequencing

According to the manufacturer's instructions (Invitrogen), total RNA was extracted from damaged spinal cord tissue using TRIzol^®^ reagent, and genomic DNA was removed using DNase I (TaKaRa). RNA quality was then determined by a 2100 Bioanalyzer instrument (Agilent), and the RNA concentration was quantified using a ND-2000 spectrophotometer (NanoDrop Technologies). Only high-quality RNA samples (OD260/280 = 1.8–2.2, OD260/230 ≥ 2.0, RIN ≥ 6.5, 28S:18S ≥ 1.0, > 10 μg) were used to construct sequencing libraries. RNA-seq transcriptome libraries were prepared using 5 μg of total RNA and the TruSeqTM RNA Sample Preparation Kit from Illumina (San Diego, CA, USA). Briefly, messenger RNA was isolated by oligo(dT) beads according to the polyA selection method, and then fragmentation buffer was added. Then, double-stranded cDNA was synthesized using the SuperScript double-stranded cDNA synthesis kit (Invitrogen, CA, USA) and random hexamer primers (Illumina). The synthesized cDNA was then end-repaired, phosphorylated, and subjected to ‘A’ base addition according to Illumina's library construction protocol. Size selection of 200–300 bp cDNA target fragments on 2% low-range ultra-agarose followed by PCR amplification using Phusion DNA polymerase (NEB) for 15 cycles was performed. After quantification by a TBS380 mini-fluometer, the paired-end RNA-seq library was sequenced with Illumina HiSeqxten (2 × 150-bp read length).

### Quality control and differential expression analysis

The original paired-end reads were trimmed, and quality control was performed with SeqPrep (https://github.com/jstjohn/SeqPrep) and Sickle (https://github.com/najoshi/sickle) with default parameters. To identify differentially expressed genes (DEGs) between two different samples, the expression level of each gene was calculated based on the number of transcripts per kilobase million (TPM). RSEM (http://deweylab.biostat.wisc.Edu/rsem/) was used to quantify gene abundance. Differential expression analysis was performed using the R statistical software package EdgeR (http://www.bioconductor.org/packages/2.12/bioc/html/edgeR.html). Statistical analysis of raw counts based on negative binomial distribution was performed using DESeq2 software. Based on certain screening criteria (*p* < 0.05 and |log2FC |> = 1), genes/transcripts with differences in expression/levels between groups were identified. In the initial data exploration phase, we performed principal component analysis (PCA) to directly calculate the coefficient of variation between groups in the R package prcomp [20].

### GO functional enrichment analysis

Five databases (NR, Swiss-Prot, Pfam, EggNOG, GO) were used to comprehensively annotate genes and transcripts. The sequences of the genes and transcripts were obtained from the NR, Swiss-Prot, and EggNOG databases with DIAMOND software. Sequence alignment was performed using the BLAST2GO and GO databases. HMMER software was used to align the sequences with entries in the Pfam database. GO enrichment analysis of genes/transcripts in certain gene sets was performed using Fisher's precise test using Goatools software. To control for false positives, the BH correction method was performed in Goatools to correct the *p* value [21]. Genes were considered significantly enriched in a GO term if the corrected *p* value (FDR) was < 0.05.

### The association network between GO functional enrichment pathways and gene targets

The DEG targets and the associated GO terms were imported into Cytoscape_v3.9.1 software [22] for visual network association analysis.

### Statistical analysis

The data are expressed as the mean ± S.D. GraphPad Prism 7.0 (GraphPad Software Inc., San Diego, CA, USA) was used for statistical analysis. Comparisons between the two groups were made using Student’s *t* test or the Mann‒Whitney test when appropriate. Multiple comparisons between groups were performed using one-way ANOVA followed by Tukey’s test. A *p* value < 0.05 was considered statistically significant.

## Results

### Atrophy of immune organs after spinal cord injury

**Fig. 2:**
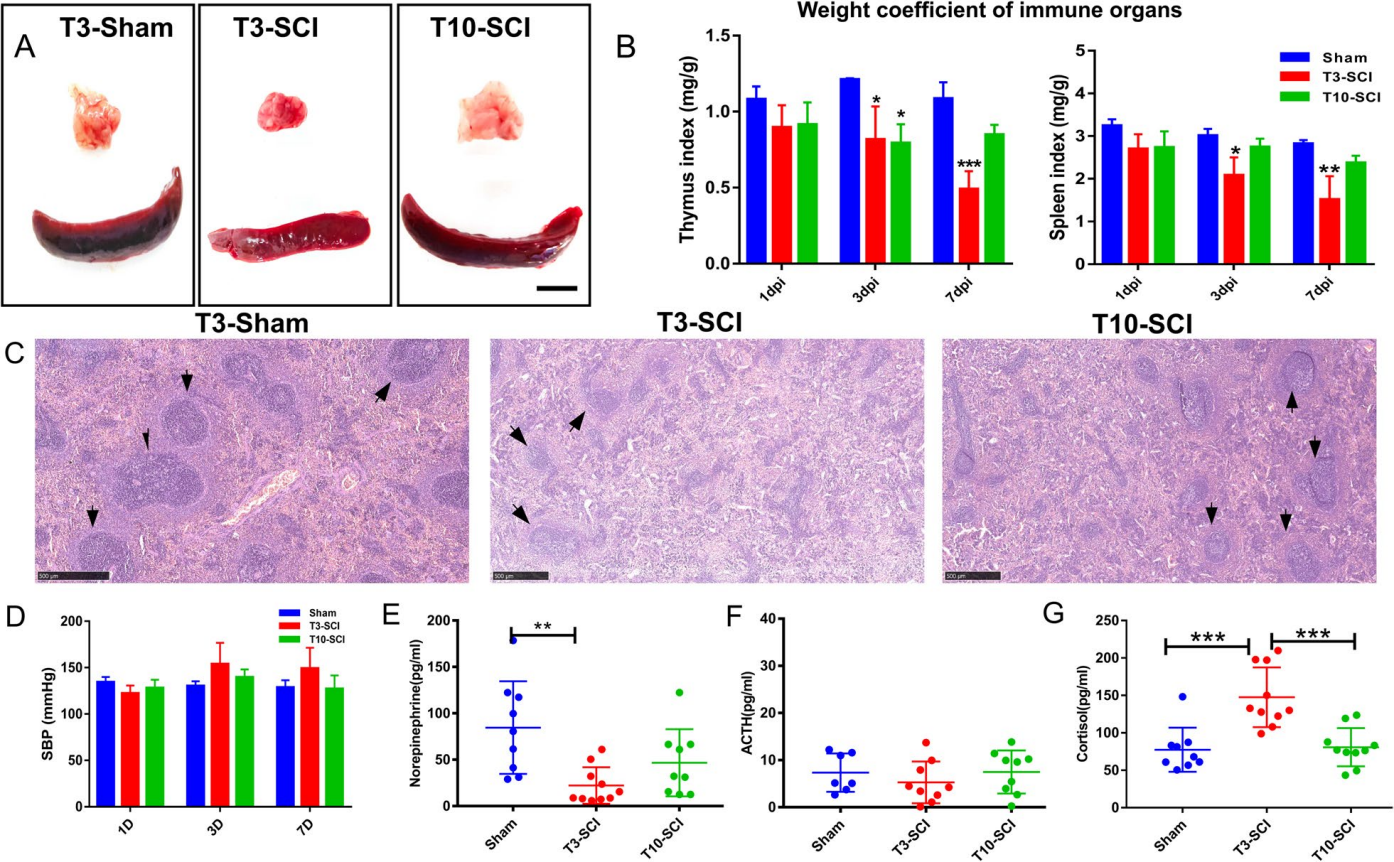
Changes in immune organ indices and physicochemical factors related to the SAM and HPA axes after SCI. **A** Compared with sham surgery and T10-SCI, T3-SCI resulted in significant shrinkage of the (top) thymus and (bottom) spleen after day 7. Scale bar, 5 mm. **B** Changes in immune organ indices after SCI. All data are presented as the mean and standard deviation (SD); *n* = 3. The left side shows the thymus index (compared with the sham group: 3 dpi: T3-SCI, *p* = 0.026; T10-SCI, *p* = 0.016; 7 dpi: T3-SCI, *p* = 0.0005). The right side shows the spleen index (compared to the sham group: 3 dpi: T3-SCI, *p* = 0.036; 7 dpi: T3-SCI, *p* = 0.0017). **C** Representative image of HE staining of the spleen after SCI. Scale bar = 500 µm. **D** Changes in SBP (mmHg) at the third time point after SCI were measured. **E**–**G** Changes in the levels of the catecholamines norepinephrine (NE), adrenocorticotropic hormone (ACTH) and cortisol (GC); *n* = 10. All data are presented as the mean and the standard deviation (SD) and were analysed using one-way and two-way ANOVA followed by Tukey’s multiple comparisons test. *p < 0.05, **p < 0.01, ***p < 0.001

Animals underwent thoracic spinal cord transection at T3 or T10. Five rats died after surgery due to surgical damage or severe bladder dysfunction; 4 of these rats underwent T10-SCI. Male rats were used in this study because the majority of SCI patients are males [15, 16]. The thymus and spleen are the main immune organs and the main hubs of innate and adaptive immune responses. We found significant atrophy of the thymus and spleen after high-level SCI (Fig. 2A). According to the immune organ indices, progressive thymus and spleen atrophy was observed in the T3-SCI group compared to the sham group; however, on day 3, rats with T10-SCI showed a decrease in the immune organ indices, which gradually recovered by day 7 (Fig. 2B). No decrease in the immune organ indices was observed in any animal after surgical laminectomy. Furthermore, we observed changes in the red pulp (RP) and white pulp (WP) of the spleen on the 7th day after injury; significant atrophy was observed in the WP of the spleen in rats with T3 injury, the boundaries were blurred, and the size of the germinal centre was reduced. The WP of the spleen was preserved in rats with T10-SCI (Fig. 2C); the WP is where lymphocyte production and differentiation occur [23]. In short, after high-level SCI, the main immune organs become atrophied, and the tissue structure of these organs is disrupted.

### Changes in sympathetic activity and expression of hormones in the HPA axis after SCI

Next, we noninvasively measured blood pressure through the rat tail artery. The aim was to detect sympathetic activity changes, which are best represented by changes in SBP, in live rats after injury to different segments of the spinal cord. We found that on days 3 and 7, the SBP of the T3-SCI group was higher than that of the other two groups, but there was no statistically significant difference among the three groups (Fig. 2D), indicating that there was no significant change in blood pressure in rats in the acute or subacute stage of injury. However, ELISA revealed a significant reduction in NE levels in the plasma of rats with T3-SCI (*p* = 0.003) (Fig. 2E), consistent with previous studies [10]. NE is the main CA produced by the adrenal medulla. Furthermore, we measured plasma cortisol levels and found significant increases in cortisol expression in the T3-SCI group compared with the sham group (*p* = 0.0001) and T10-SCI group (*p* = 0.0002) (Fig. 2G). However, no significant differences in ACTH levels in plasma were observed among the three groups (Fig. 2F). Cortisol is produced in the zona fasciculata of the adrenal cortex and is the effector hormone of the HPA axis [24]. In summary, in the subacute phase after high-level SCI, the expression of two main effector hormones of the adrenal gland is reduced, the expression of a third effector hormone is significantly increased, and endocrine dysfunction may be involved in the impairment of peripheral immunity.

### T3 SCI results in significant inhibition of the spleen immune response

The experimental results revealed immune organ atrophy after high-level SCI, alterations in adrenal-mediated endocrine hormone expression, and structural changes. Next, to further understand the pathogenesis of SCI-IDS, we analysed gene expression changes in neural (hypothalamic), endocrine (adrenal) and immune (spleen) tissues and organs of the same animal after injury to different spinal cord segments through unbiased transcriptomics and explored the DEGs in depth. RNA-seq of 3 biological replicates from the sham group and 5 biological replicates from the other two groups was performed. Quality control data for samples from all groups were obtained for bioinformatics analysis (Additional file 1). Sample-to-sample PCA was performed prior to all genome analyses to exclude outlier DEGs (Additional file 2).

**Fig. 3:**
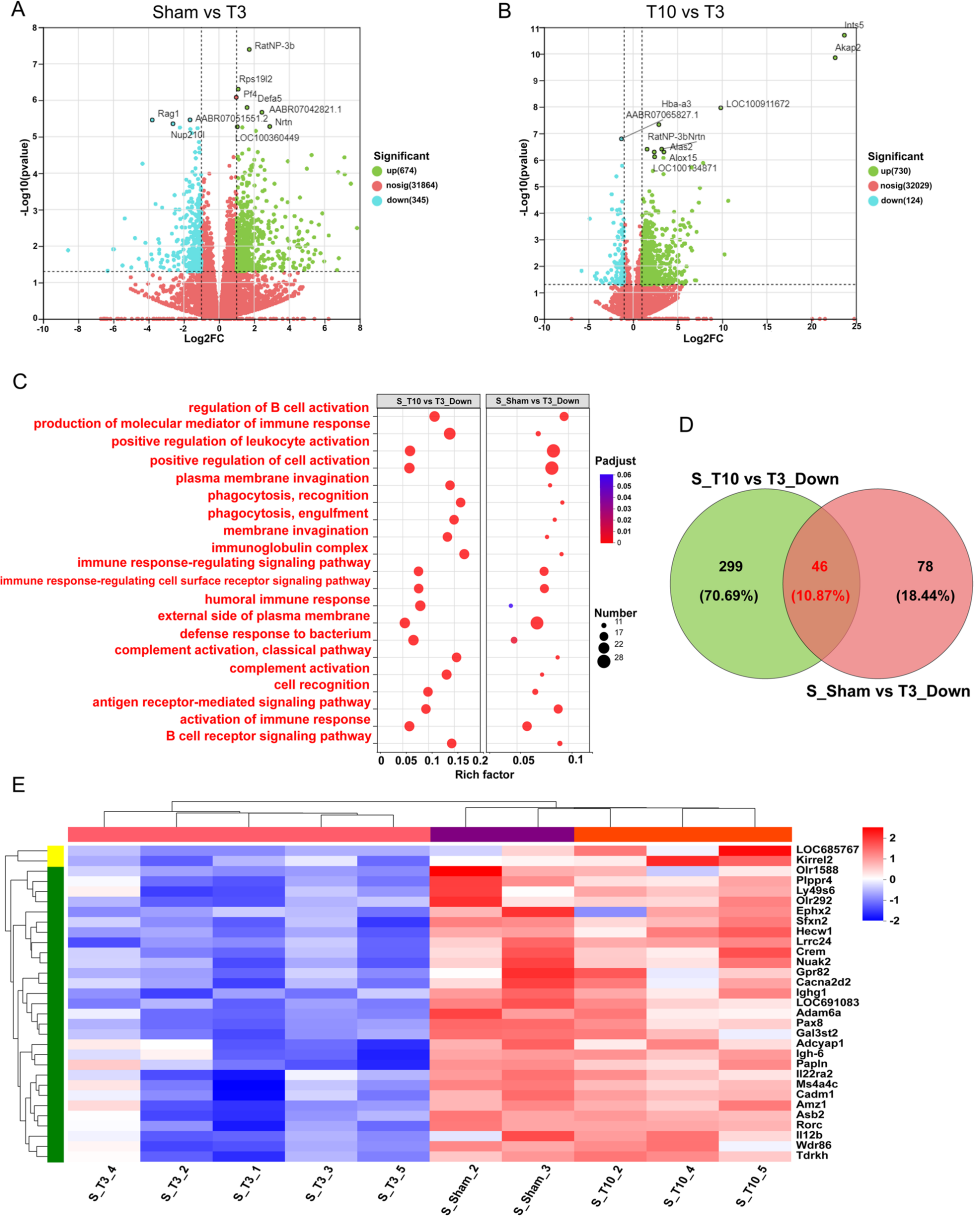
T3-SCI completely suppresses splenic immunity. **A** Volcano plot of DEGs between the sham group and the T3-SCI group; green indicates upregulated DEGs, and red indicates downregulated DEGs. **B** Volcano plot of DEGs between the T10-SCI group and the T3-SCI group. Green indicates upregulated DEGs, and red indicates downregulated DEGs. **C** Top 20 commonly enriched GO terms for DEGs between the sham group and the T3-SCI group and between the T10-SCI group and the T3-SCI group. **D** Venn diagram analysis of downregulated DEGs between the sham group and the T3-SCI group and between the T10-SCI group and the T3-SCI group. The red numbers represent downregulated DEGs specific to rats in the T3-SCI group for both comparisons. **E** Cluster analysis of downregulated DEGs in the T3-SCI group according to (**D**) Venn diagram analysis; blue represents downregulated DEGs, and red indicates upregulated DEGs

Figure 3A shows a volcano plot of the DEGs in the spleen between the sham and T3-SCI groups identified by RNA-seq; 1019 genes were differentially expressed between the two groups, with 674 genes being upregulated and 345 genes being downregulated in the T3-SCI group. The DEGs between the T3-SCI and T10-SCI groups are shown in Fig. 3B; 854 genes were differentially expressed between the two groups, with 730 genes being upregulated and 124 genes being downregulated in the T3-SCI group.

To further explore the regulatory function of the spleen after SCI in the T3-SCI group, GO enrichment analysis of the DEGs between the groups was performed, and the GO terms in which the DEGs were significantly enriched were identified. Figure 3C shows the top 20 results of GO-enriched terms in rat spleen DEGs after T3 injury relative to the Sham group and the T10-SCI group. Surprisingly, the top 20 enriched GO terms for the downregulated DEGs in the T3-SCI group included immunomodulation-related terms, such as "regulation of B-cell activation", "production of molecular mediator of immune response", "positive regulation of leukocyte activation", "immunoglobulin complex", "humoral immune response", "antigen receptor-mediated signaling pathway", "activation of immune response" and "B-cell receptor signaling pathway" (Fig. 3C). This indicates that the overall immune response of the spleen, especially the participation of B cells in humoral immunity, was decreased after T3-SCI; this is consistent with the atrophy of spleen RP, which is involved in antibody production, observed in the previous experiment. In addition, GO enrichment analysis of the DEGs upregulated in the T3-SCI groups did not reveal any significantly enriched terms. A total of 46 DEGs were specifically expressed in the spleen in the T3-SCI group according to Venn diagram analysis, and unannotated genes were eliminated (Fig. 3D). Among them, 31 key DEGs were clearly clustered in the T3-SCI group and were involved in fully inhibiting the immune function of the spleen (Fig. 3E).

In summary, the results of this transcriptomic analysis showed that the immune function of the spleen was fully inhibited after T3-SCI and further demonstrated that peripheral immunosuppression is significantly related to the level of SCI.

### DEGs in the hypothalamus after T3-SCI

**Fig. 4:**
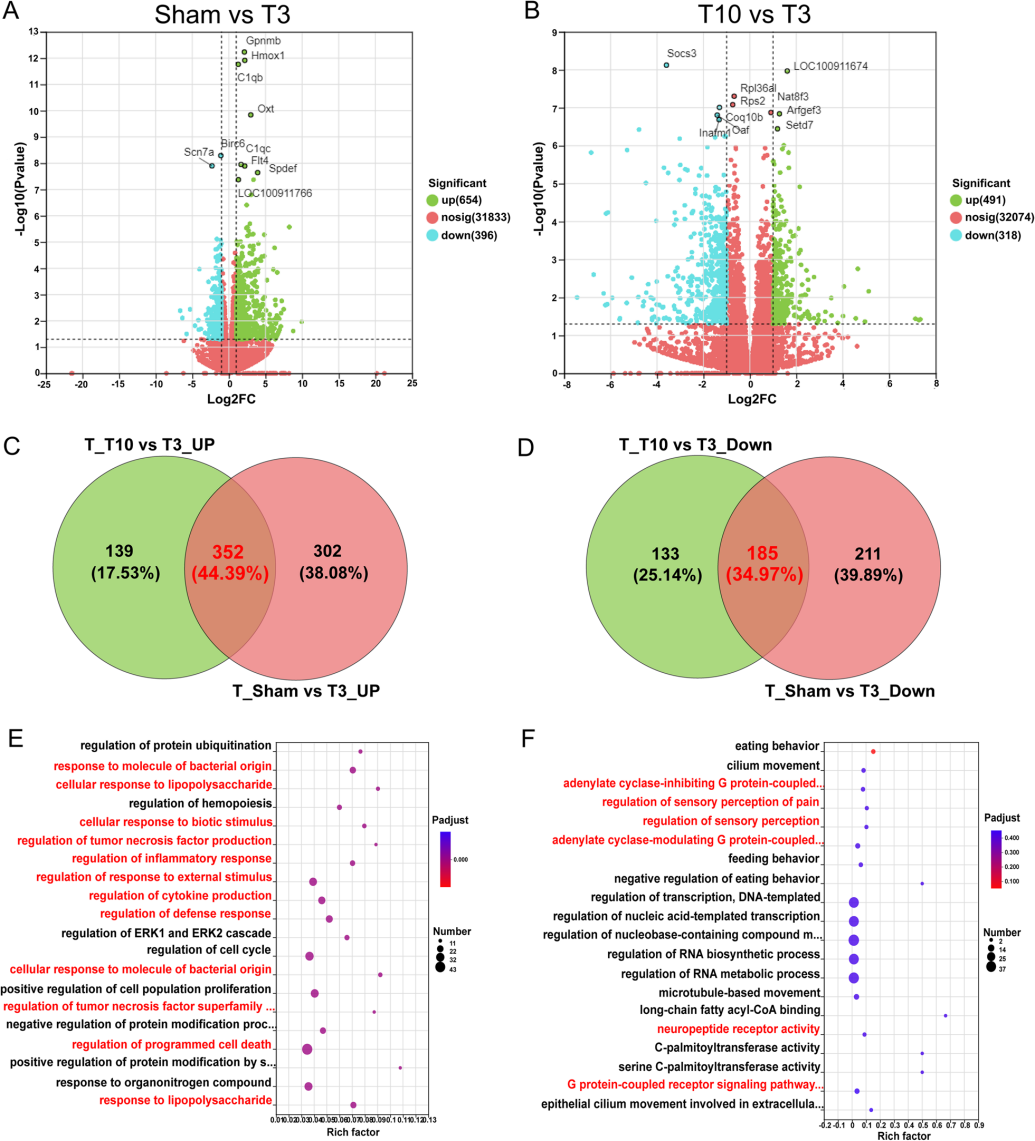
Changes in gene expression in the hypothalamus after T3-SCI. **A** Volcano plot of DEGs between the sham group and the T3-SCI group; green indicates upregulated DEGs, and red indicates downregulated DEGs. **B** Volcano plot of DEGs between the T10-SCI group and the T3-SCI group. Green indicates upregulated DEGs, and red indicates downregulated DEGs. **C** Venn diagram analysis of common upregulated DEGs between the sham group and the T3-SCI group and between the T10-SCI group and the T3-SCI group. The red numbers represent upregulated DEGs specific to rats in the T3-SCI group in both comparisons. **D** Venn diagram analysis of common downregulated DEGs between the sham group and the T3-SCI group and between the T10-SCI group and the T3-SCI group. The red numbers represent downregulated DEGs specific to rats in the T3-SCI group in both comparisons. **E** GO enrichment analysis of the upregulated DEGs in the T3-SCI group identified by Venn diagram analysis (**C**). **F** The top 20 enriched GO terms for the downregulated DEGs in the T3-SCI group identified by Venn diagram analysis (**D**)

Based on splenic RNA-seq, the increased susceptibility to infection by immunodeficiency syndrome due to SCI depends on the level of the lesion. To further investigate immunodeficiency in the T3-SCI group, we performed RNA-seq of hypothalamic tissue. The hypothalamus is a central region that integrates information and regulates autonomic and endocrine homeostasis [12]. It is also the central regulatory region for providing negative feedback to the HPA axis, and studies have shown that SCI-IDS is mediated by central preganglionic injury [6, 7]. Figure 4A shows a volcano plot of the DEGs in the hypothalamus between the sham and T3-SCI groups; 1048 genes were differentially expressed between the two groups, with 654 genes being upregulated and 396 genes being downregulated in the T3-SCI group. Figure 4B shows a volcano plot of DEGs between the T3-SCI and T10-SCI groups; 809 genes were differentially expressed between the two groups, with 491 genes being upregulated and 318 genes being downregulated in the T3-SCI group.

Similarly, we further explored the hypothalamic-specific DEGs in the T3-SCI group. First, we identified common DEGs between the T3-SCI group and the sham group and between the T3-SCI group and the T10-SCI group by Venn analysis; there were a total of 352 upregulated DEGs (Fig. 4C) and 185 downregulated DEGs (Fig. 4D). The top 20 enriched GO terms were obtained; the common upregulated DEGs were mainly associated with inflammatory pathways, such as "response to molecule of bacterial origin", "cellular response to lipopolysaccharide", "cellular response to biotic stimulus", "regulation of tumor necrosis factor production”, “regulation of inflammatory response”, “regulation of cytokine production”, “cellular response to molecule of bacterial origin”, “regulation of tumor necrosis factor superfamily cytokine production”, “regulation of programmed cell death”, and “response to lipopolysaccharide” (Fig. 4E; Additional file 3). Interestingly, the downregulated DEGs were enriched in pathways associated with inhibitory Gi-mediated G protein-coupled receptor (Gi-GPCR) neurons and neuropeptide changes, such as "adenylate cyclase-inhibiting G protein-coupled receptor signaling pathway", "regulation of sensory perception of pain", "regulation of sensory perception”, “adenylate cyclase-modulating G protein-coupled receptor signaling pathway”, “neuropeptide receptor activity” and “G protein-coupled receptor signaling pathway, coupled to cyclic nucleotide second messenger" (Fig. 4F; Additional file 4).

In conclusion, after high-level SCI, neuroinflammation spreads to higher spinal cord levels, while uncontrolled and progressive central inflammation may mediate neural network remodelling of higher centres, as manifested by suppression of inhibitory Gi-mediated GPCR-expressing neuron activity, leading to enhanced excitatory neurotransmission in the brain or CNS hyperconnectivity.

### T3-SCI promotes the spread of neuroinflammation to the hypothalamic PVN and hippocampal DG above the spinal cord

To verify the secondary response to inflammation above the spinal cord after T3-SCI, we examined glial cells in the paraventricular nucleus (PVN) of the hypothalamus and the dentate gyrus (DG) of the hippocampus. Microglia, which express ionized calcium binding adapter molecule 1 (IBA1) [25], are the most sensitive neuroimmune cells in the CNS, and microglial activation and morphological changes are the hallmarks of neuroinflammation. As the most abundant glial cells in the brain, astrocytes, which express glial fibrillary acidic protein (GFAP), play the role of CNS “housekeepers" by providing metabolic and nutritional support; regulating synaptic synaptogenesis, ion homeostasis, and neurotransmitter buffering; maintaining the integrity of the BBB; and promoting neural network activity [26]. In addition, postsynaptic density (PSD) 95, which is a key protein for postsynaptic signal transduction and integration, mainly at mature excitatory glutamatergic synapses, is located in the postsynaptic membrane in the CNS [27].

**Fig. 5:**
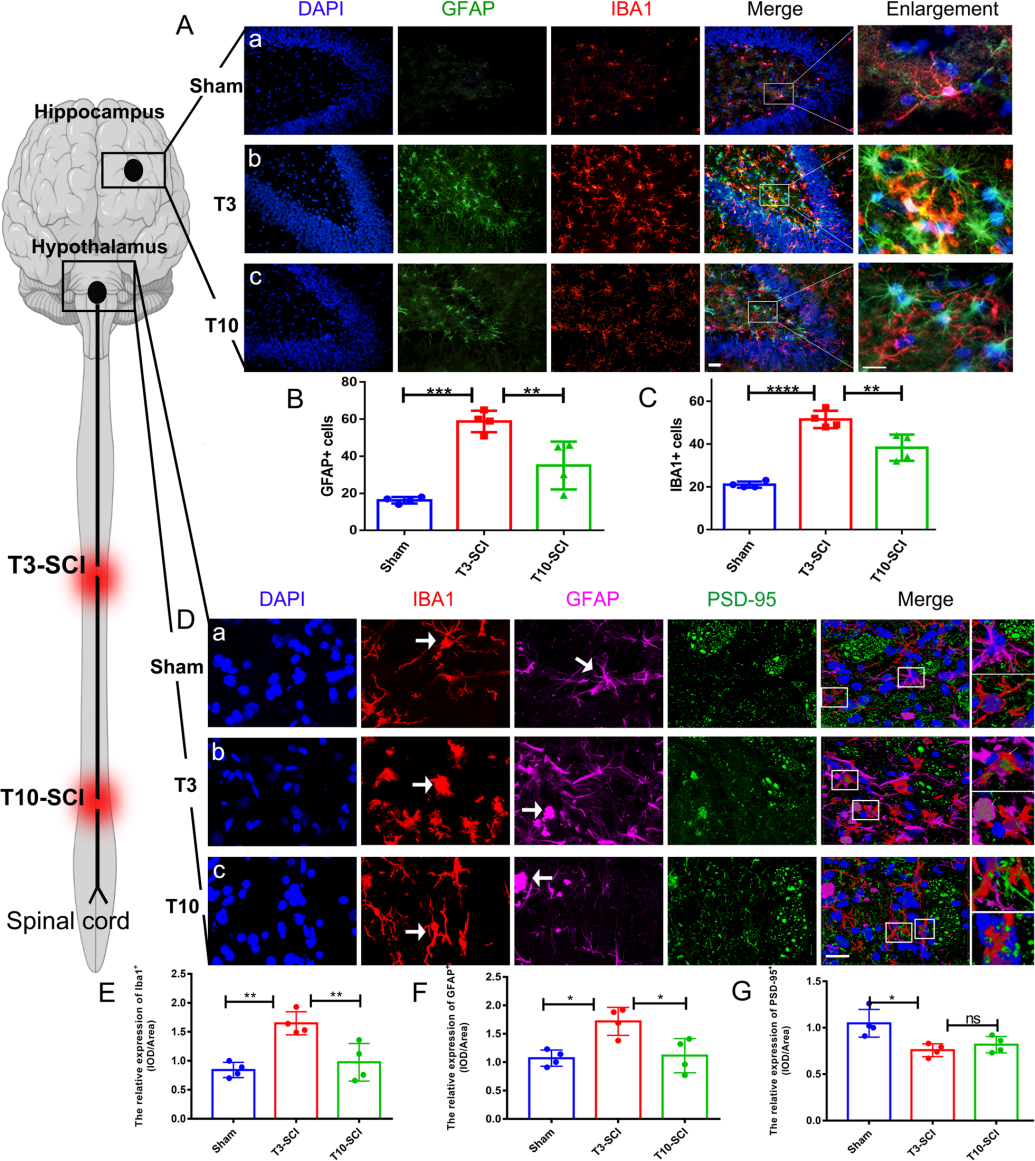
Post-SCI neuroinflammation in the hypothalamic PVN and hippocampal DG regions. **A** Representative images showing GFAP and IBA1 immunofluorescence staining in the hippocampal DG after SCI. Scale bar = 200 µm. A is the sham group, b is the T3-SCI group, and c is the T10-SCI group. **B**, **C** Quantitative analysis of the data in Figure A; all data are presented as the mean and standard deviation (SD); *n* = 4. **B** Number of GFAP + positive cells; (**C**) number of IBA1 + positive cells. **C** Representative high-magnification images of GFAP, IBA1, and PSD95 immunofluorescence staining in the hippocampal DG region after SCI. Scale bar = 50 µm. A is the sham group, b is the T3-SCI group, and c is the T10-SCI group. One-way and two-way ANOVA followed by Tukey’s multiple comparison test were used to analyse differences between groups. *p < 0.05, **p < 0.01, ***p < 0.001

Next, we performed immunofluorescence staining of the DG of the hippocampus; IBA1 staining in the DG revealed the presence of quiescent microglial with a scattered, branch-like morphology in the normal sham group. IBA1 expression was increased in the T10-SCI group, but the IBA1-stained cells in the T10-SCI group were morphologically similar to those in the sham group, indicating the presence of proliferative microglia. In contrast, in the T3-SCI group, the number of IBA1-positive cells in the DG was obviously increased, the somata were enlarged, the cell branches were shortened, and the cells showed an amoeboid-like morphology, indicating that microglia were activated. GFAP expression showed a similar change, indicating astrocyte overactivation (Fig. 5A). Statistical analysis revealed that the numbers of GFAP + cells, IBA1 + cells and GFAP + /IBA1 + double-positive cells were significantly increased in the T3-SCI group compared with the sham group and the T10-SCI group (Fig. 5B, C) and that the colocalization of microglia and astrocytes was increased, indicating increased interactions and tight connections between the two cell types. In summary, we found that high-level (T3) SCI leads to exacerbation of inflammation in the hippocampal DG region associated with neurogenesis.

Next, we further assessed neuroinflammation in the PVN by evaluating changes in the expression of the postsynaptic membrane protein PSD-95 via triple immunofluorescence for GFAP, IBA1, and PSD-95 (Fig. 5D). The number of activated GFAP + or IBA1 + cells in the PVN was higher in the T3-SCI group than in the other two groups (Fig. 5E, F), with the number of IBA1-labelled microglia being especially increased, further indicating a broad inflammatory response throughout the upper spinal cord after high-level SCI. This confirms the abovementioned findings of GO enrichment analysis showing that inflammatory signalling pathway activity was increased. Interestingly, despite the exacerbation of inflammation, the expression of the PSD95 protein was significantly decreased in the T3-SCI group compared with the sham group (*p* < 0.05), while there was a trend toward downregulation of PSD95 in the T3-SCI group compared with the T10-SCI group, the difference was not significant (Fig. 5G). This suggests that post-SCI central inflammation may be negatively correlated with PSD95-mediated neurotransmission and that inflammation-mediated excitation inhibits neuronal Gi-GPCR expression, which is consistent with what was revealed by GO enrichment analysis of the downregulated DEGs.

In summary, we found that high-level SCI leads to secondary peripheral immunodeficiency and exacerbation of an uncontrollable inflammatory response in the CNS, while inflammation and dysregulation of neurotransmitters in the hypothalamus, which plays an important role in the HPA axis, may be involved in the central regulation of secondary immunodeficiency after SCI.

### T3-SCI mediates widespread dispersion of spinal canal neuroinflammation

**Fig. 6:**
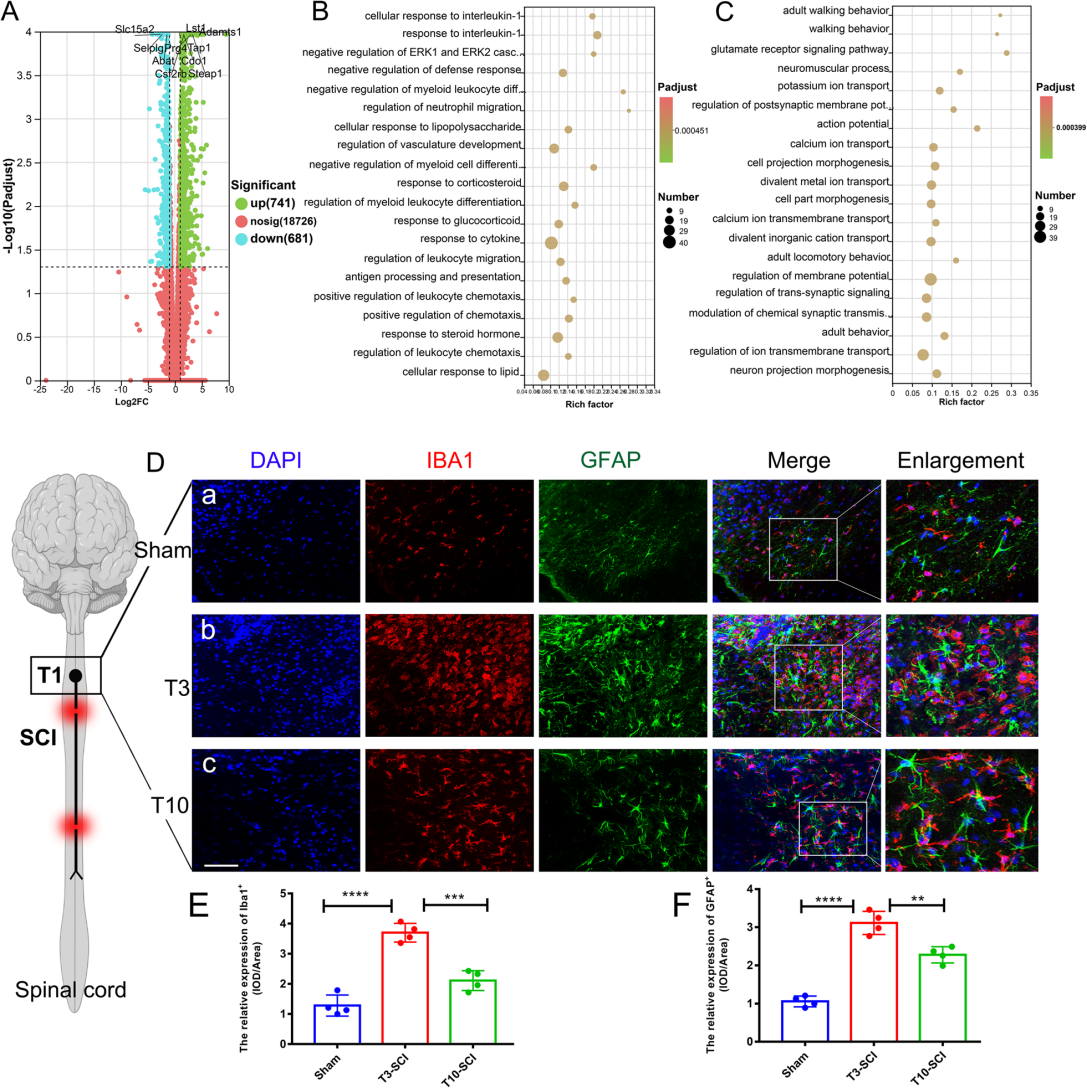
Differential gene expression in the T3 spinal cord and neuroinflammation after T3-SCI. **A** Volcano plot of DEGs between the sham group and the T3-SCI group; green indicates upregulated DEGs, and red indicates downregulated DEGs. **B** GO enrichment analysis of upregulated DEGs in the spinal cord after T3-SCI. **C** GO enrichment analysis of downregulated DEGs in the spinal cord after T3-SCI. **D** Representative images showing GFAP and IBA1 immunofluorescence staining in the T1 spinal cord after SCI (a is the sham group, b is the T3-SCI group, and c is the T10-SCI group). Scale bar = 100 µm. **E** Quantitative analysis of the IBA1 + expression intensity in Figure **A**; all data are presented as the mean and standard deviation (SD); *n* = 4. **F** Quantification of the GFAP staining intensity in Figure **A**. All data are presented as the mean and standard deviation (SD); *n* = 4. One-way and two-way ANOVA followed by Tukey’s multiple comparisons test were used to analyse differences between groups. *p < 0.05, **p < 0.01, ***p < 0.001

We have previously published a rapid secondary inflammatory response following SCI [25]. Here, to further explore the spread of inflammation from the spinal cord to the PVN and hippocampus, we performed RNA-seq of the injured T3 spinal cord. Figure 6A shows a volcano plot of the DEGs in injured spinal cord tissues from the sham and T3-SCI groups; there were 1422 DEGs, with 741 genes being upregulated and 681 genes being downregulated in the T3-SCI group.

Subsequently, the functions of these DEGs were analysed by GO enrichment analysis. The top 20 enriched biological process (BP) terms in which the DEGs were enriched were identified. Among them, "cellular response to interleukin-1", "response to interleukin-1", "regulation of neutrophil migration", "cellular response to lipopolysaccharide", "response to corticosteroid", "regulation of myeloid leukocyte differentiation", "response to glucocorticoid", "response to cytokine", "regulation of leukocyte migration", "antigen processing and presentation", "positive regulation of leukocyte chemotaxis" and "response to steroid hormone" had the highest regulatory enrichment rates (Fig. 6B; Additional file 5), and the results showed that almost all of the GO terms were associated with neuroinflammation and the response to cortisol. The enriched GO terms for the downregulated DEGs mainly included "glutamate receptor signalling pathway", "regulation of postsynaptic membrane potential", "calcium ion transmembrane transport", "regulation of transsynaptic" signaling", "modulation of chemical synaptic transmission", "regulation of ion transmembrane transport" and "neuron projection morphogenesis" (Fig. 6C; Additional file 6).

In summary, it is illustrated here that rapid and widespread neuroinflammation is the main pathological change after SCI; this change leads to neuronal death and blocks neurotransmitter transmission. Interestingly, the transcriptome of the injured T3 spinal cord was very similar to that of the hypothalamus, and the GO terms in which the DEGs were enriched were very similar. Then, we performed double immunofluorescence staining for GFAP and IBA1 in injured upper spinal cord tissue (T1) and found obvious infiltration of various types of activated microglia/macrophages in the spinal cord close to the site of injury (T3) (Fig. 6D–E) and concomitant extensive astrocyte reactivity (Fig. 6D, F); in the figures, astrocyte reactivity in the T1 spinal cord is shown in the T10-SCI group, in which T1 was further from the injury site than in the T3-SCI group. GFAP and IBA1 staining was still strong. In summary, it is further concluded that extensive enhancement of neuroinflammation plays a central role in SCI pathogenesis.

### DEGs in the adrenal glands after T3-SCI

**Fig. 7:**
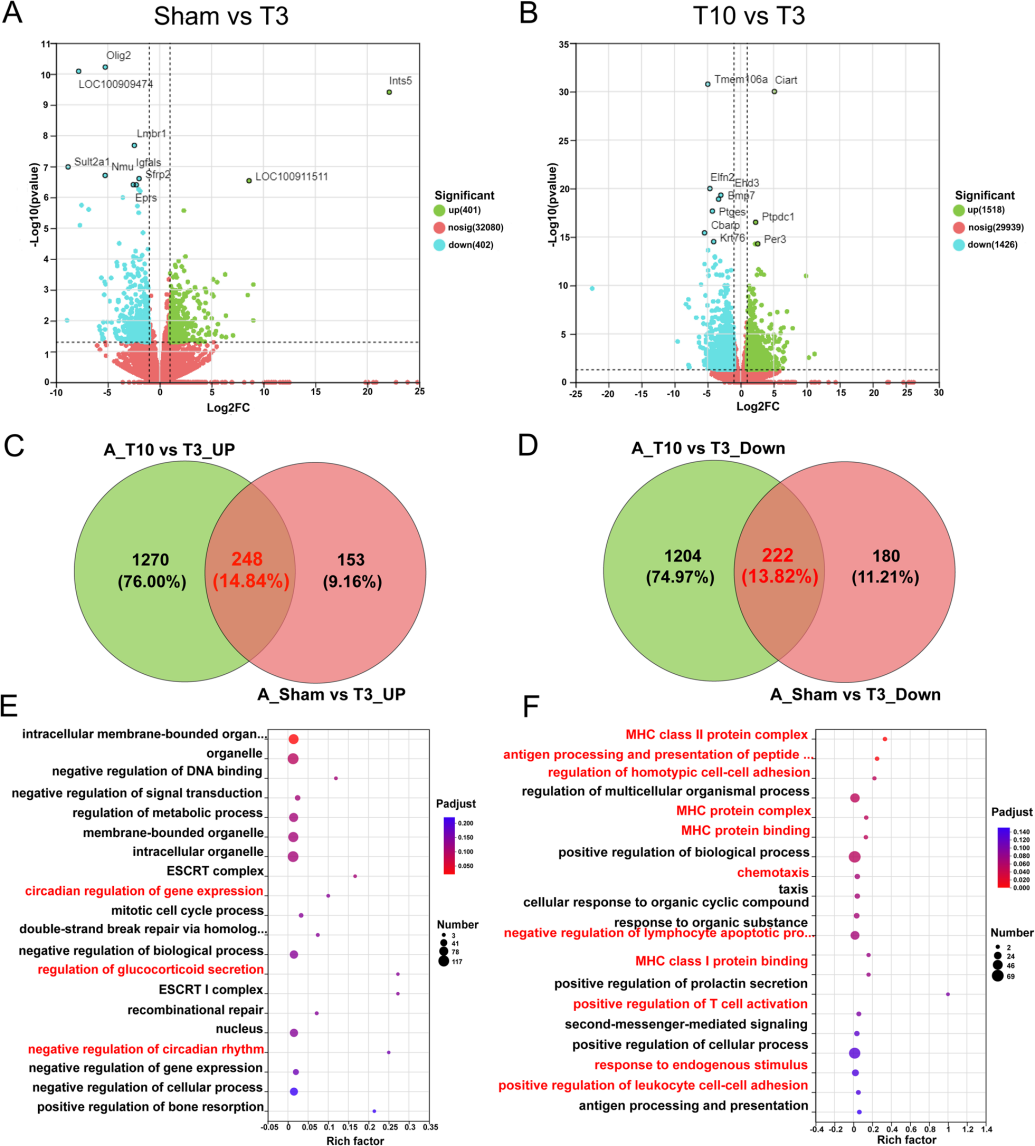
Changes in gene expression in the adrenal glands after T3-SCI. **A** Volcano plot of DEGs between the sham group and the T3-SCI group; green indicates upregulated DEGs, and red indicates downregulated DEGs. **B** Volcano plot of DEGs between the T10-SCI group and the T3-SCI group. Green indicates upregulated DEGs, and red indicates downregulated DEGs. **C** Venn diagram analysis of common upregulated DEGs between the sham group and the T3-SCI group and between the T10-SCI group and the T3-SCI group. The red numbers represent upregulated DEGs in the adrenal glands specifically in rats in the T3-SCI group in the two comparisons. **D** Venn diagram analysis of common downregulated DEGs between the sham group and the T3-SCI group and between the T10-SCI group and the T3-SCI group. The red numbers represent downregulated DEGs in the adrenal glands specifically in rats in the T3-SCI group in the two comparisons. **E** GO enrichment analysis of the upregulated DEGs in the T3-SCI group identified by Venn diagram analysis (**C**). **F** The top 20 enriched GO terms for the downregulated DEGs in the T3-SCI group identified by Venn diagram analysis (**C**)

The adrenal glands are important endocrine organs for maintaining homeostasis, and proper adrenal gland function protects against infection after SCI [10]. Subsequently, differential gene expression in the adrenal glands after T3-SCI was assessed to elucidate the mechanism underlying SCI-IDS. Figure 7A shows a volcanic plot of the DEGs in the adrenal glands between the sham and T3-SCI groups identified by RNA-seq; 803 genes were differentially expressed between the two groups, with 401 genes being upregulated and 402 genes being downregulated in the T3-SCI group. Figure 7B shows a volcano plot of DEGs between the T3-SCI and T10-SCI groups; there were 2944 DEGs between the two groups, with 1518 genes being upregulated and 1426 genes being downregulated in the T3-SCI group. Similarly, we further explored adrenal gland-specific changes in gene expression in the T3-SCI group. First, the common DEGs in adrenal tissue between the T3-SCI group and the sham group and between the T3-SCI group and the T10-SCI group were identified by Venn diagram analysis; there were 248 common upregulated (Fig. 7C) and 222 common downregulated (Fig. 7D) DEGs. The top 20 GO terms in which the common DEGs were enriched were determined; the common upregulated DEGs were mainly enriched in terms associated with cortisol secretion and circadian rhythm changes, such as "circadian regulation of gene expression", "regulation of glucocorticoid secretion" and "negative regulation of circadian rhythm" (Fig. 7E; Additional file 7). This indicates that circadian rhythm disorder, which is closely related to cortisol secretion, was more severe in the T3-SCI group than in the sham group or the T10-SCI group. Moreover, the increase in cortisol expression was indeed more significant in the T3-SCI group (Fig. 2G).

Interestingly, the downregulated DEGs in the adrenal gland after T3 injury were significantly enriched in terms associated with major histocompatibility complexes (MHCs), especially MHC-II class immune responses, such as "MHC class II protein complex", "antigen processing and presentation of peptide or polysaccharide antigen via MHC class II", "MHC protein complex", "MHC protein binding", "chemotaxis", "MHC class I protein binding", "positive regulation of T-cell activation" and "antigen processing and presentation" (Fig. 7F; Additional file 8). MHC-II is mostly located on antigen-presenting cells, such as macrophages, and uses MHC to prompt helper T cells to initiate an immune response [28]. In summary, T3 injury not only led to the marked impairment of splenic immune function but also inhibited the adaptive immune response mediated by adrenal MHC-II.

### Changes in the distribution of postganglionic sympathetic nerves in adrenal tissue after SCI

Structurally, the adrenal glands are divided into the cortex and medulla; moreover, the adrenal are important effector organs of the HPA axis and the SAM axis. An increasing number of scholars believe that IDS, in which sympathetic innervated chromaffin cells secrete NE to promote apoptosis of immune cells [29], is indirectly caused by adrenal gland impairment after SCI [10]. In addition, excess cortisol strongly inhibits the maturation and differentiation of immune cells [30].

**Fig. 8:**
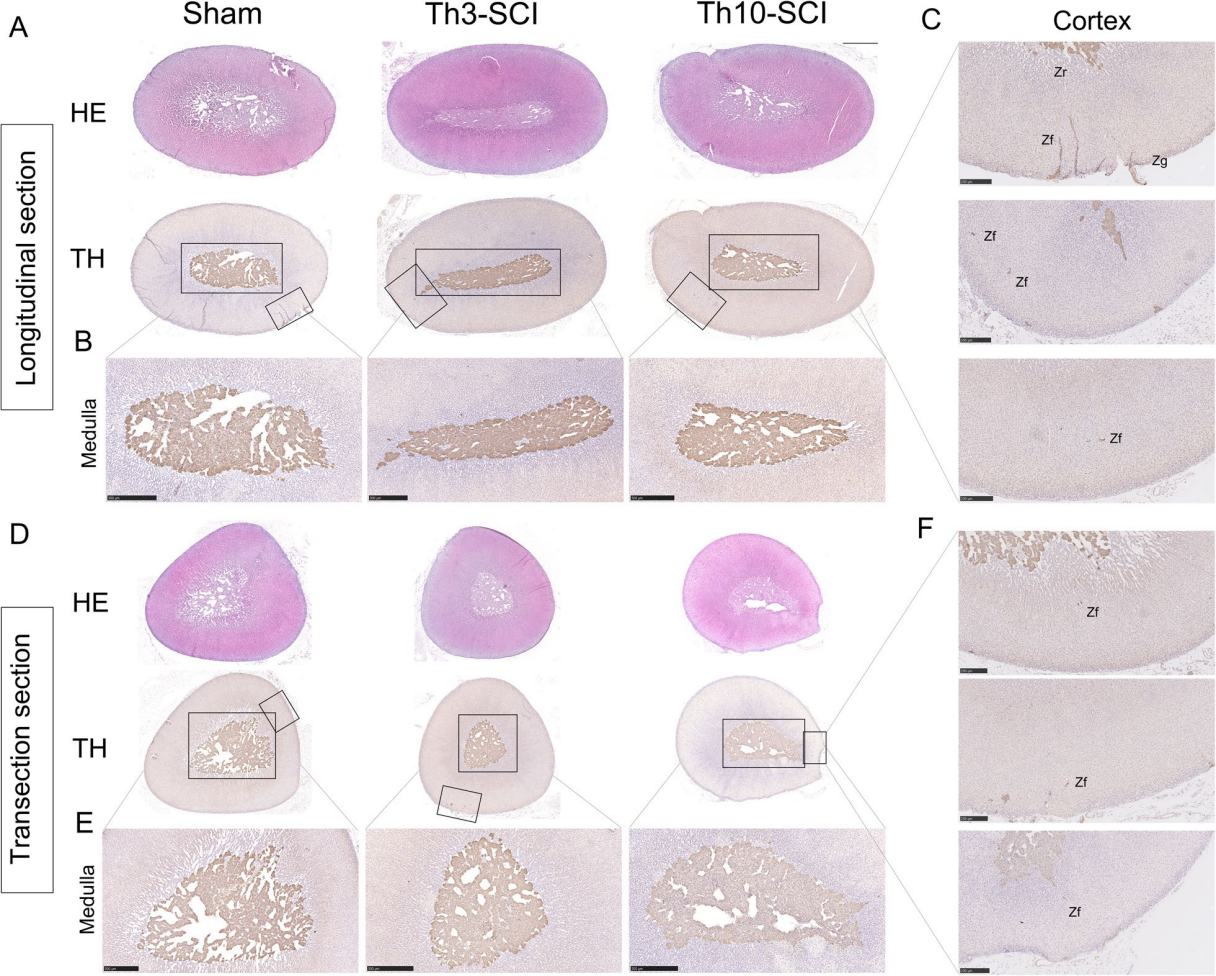
Changes in adrenal gland histomorphology and TH distribution after SCI. **A** Graph of the intensity of HE staining and immunohistochemical staining for TH in longitudinal sections of adrenal gland tissue on day 7 after SCI. *n* = 3. Scale bar = 1 mm. **B** TH distribution and expression in the medulla (m) in longitudinal sections of the adrenal gland. Scale bar = 500 µm. **C** TH distribution and expression in longitudinal seconds of the adrenal cortex (c). Scale bar = 250 µm. **D** Graph of the intensity of HE staining and immunohistochemical staining for TH in cross-sections of the adrenal gland surface on day 7 after SCI. *n* = 3. Scale bar = 1 mm. **E** TH distribution and expression in cross-sections of the adrenal medulla (m). Scale bar = 500 µm. **F** TH distribution and expression in the cross-sections of the adrenal cortex (c). Scale bar = 250 µm. *Zr* zona reticularis, *Zf* zona fasciculata, *Zg* zona glomerulosa

Next, we observed structural changes in longitudinal sections (Fig. 8A) and cross-sections (Fig. 8D) of adrenal tissue and changes in the distribution of tyrosine hydroxylase (TH), an enzyme involved in norepinephrine synthesis, in the sympathetic ganglion on day 7 after injury by morphological analysis and immunohistochemistry. Analysis of longitudinal adrenal gland sections from rats in the T3-SCI group revealed that the medulla was tightly arranged (Fig. 8B, E), TH expression was more compact, and TH + neuronal pseudopodia extended to cortical regions (Fig. 8C, F), including the zona reticularis, zona fasciculata, and zona glomerulosa. Importantly, this shows that the distribution of postganglionic sympathetic nerves in adrenal tissue was not been significantly affected after SCI and that there was still a large number of postganglionic neurons innervating cortical regions, which also provides a basis for the autonomous regulation of adrenal gland-mediated GC secretion after injury.

### Networks linking enriched pathways and gene targets in the hypothalamus and adrenal glands

**Fig. 9:**
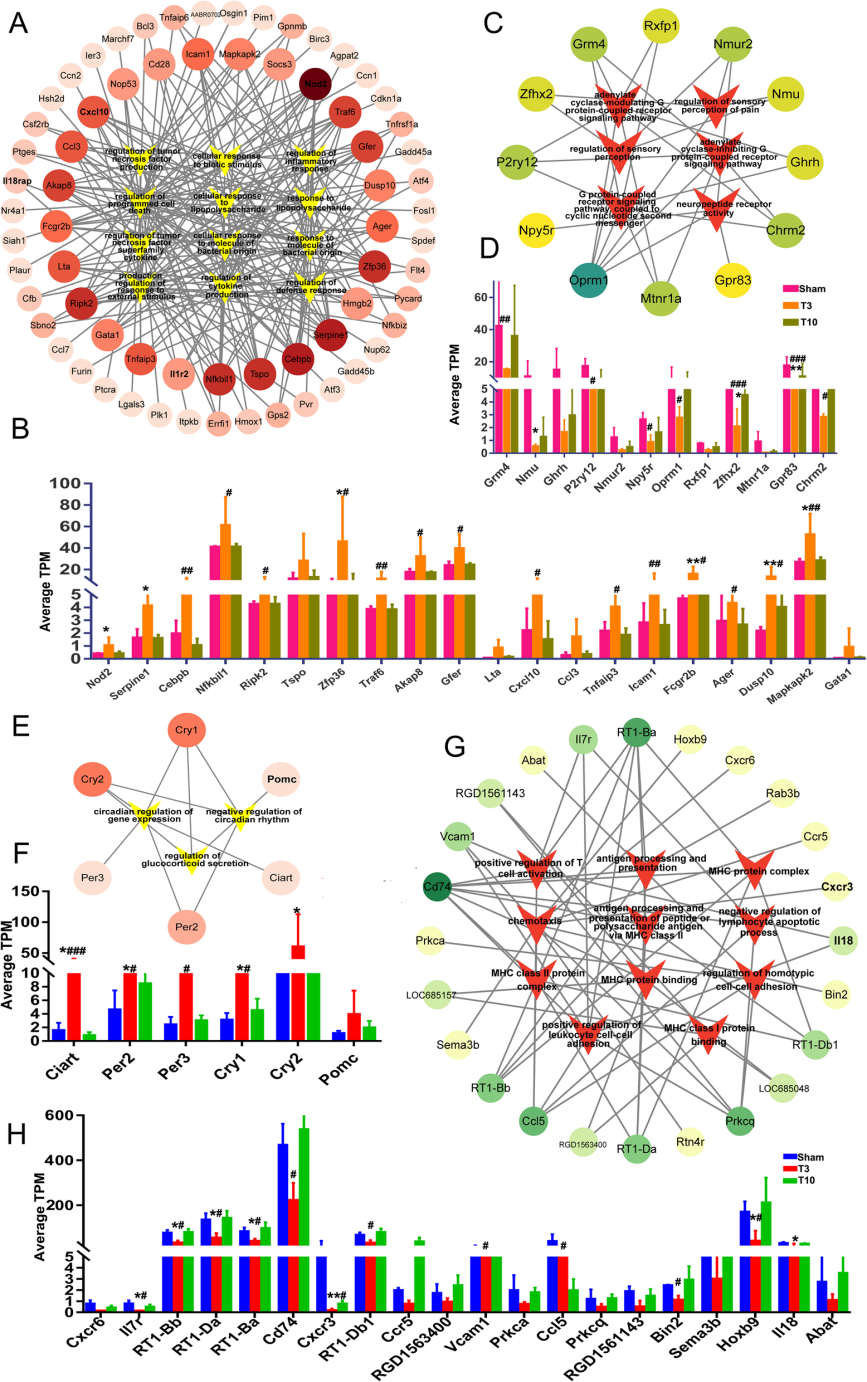
GO enrichment analysis of the association network between gene targets in the hypothalamus and adrenal glands after T3-SCI. (**A**, **B**) (**A**) Targets of upregulated genes associated with enriched GO pathways in the hypothalamus after T3-SCI. **B** Expression of the top 20 hub genes most closely associated with the GO pathways (in TPM). (**C**, **D**) **C** Targets of downregulated genes associated with enriched GO pathways in the hypothalamus after T3-SCI. **D** Expression of the top 20 hub genes most closely associated with the GO pathways (in TPM). (**E**–**F**) **E** Targets of upregulated genes associated with enriched GO pathways in the adrenal glands after T3-SCI. **F** Expression of the top 20 hub genes most closely associated with the GO pathways (in TPM). (**G**, **H**) **G** Targets of downregulated genes associated with the enriched GO pathways in the hypothalamus after T3-SCI. **H** Expression of the top 20 hub genes most closely associated with the GO pathways (in TPM). The T3-SCI group compared to the sham group: *p < 0.05, **p < 0.01, ***p < 0.001; the T3-SCI group compared to the T10-SCI group: #*p* < 0.05, ##*p* < 0.01, ###*p* < 0.001

To investigate the relationship between the pathways enriched in high-level SCI and DEG targets, we constructed and visualized PPI networks using the network visualization software Cytoscape (v3.9.1). Here, we used enriched GO terms for the DEGs in the hypothalamic and adrenal glands as nodes of interest and the gene targets associated with them as edges and constructed a network map to reveal the hub genes in the pathway. The darker the node colour is, the higher the degree centrality (DC), indicating that the correlation between the gene target and the pathway of interest is more significant (Fig. 9A, C, E, G). The 20 DEGs with the highest DC were considered hub genes; Fig. 9D, F, H show the change in the TPM of these genes, which reflects the change in their expression.

The significantly upregulated genes in the hypothalamus, i.e., *Nod2, Serpine1, Cebpb, Nfkbil1, Ripk2, Zfp36, Traf6, Akap8, Gfer, Cxcl10, Tnfaip3, Icam1, Fcgr2b, Ager, Dusp10,* and *Mapkapk2*, were enriched in 12 inflammation-related pathways (Fig. 9A, B). The downregulated hub genes, i.e., *Grm4, Nmu, P2ry12, Npy5r, Oprm1, Zfhx2, Gpr83,* and *Chrm2*, were enriched in 6 pathways related to changes in neuronal Gi-GPCR and neuropeptide expression (Fig. 9C, D). GO enrichment analysis showed that the upregulated hub genes in the adrenal glands, i.e., *Ciart, per2, per3, cry1,* and *cry2*, were significantly enriched in three pathways related to cortisol secretion and circadian rhythm changes and the related hub genes (Fig. 9E, F). The downregulated hub genes, i.e., *IL7r, rt1-bb, rt1-bb1, rt1-da, rt1-ba, cd74, cxcr3, vcam1, ccl5, bin1,* and *IL8*, were enriched in 11 pathways associated with the MHC-mediated immune response (Fig. 9E, F).

## Discussion

Immunodeficiency after SCI leads to increased susceptibility to infection and severely affects the recovery of function [5–8]. However, there is still a lack of systematic understanding of SCI-IDS. In the current study, we found that the immune organs showed persistent atrophy after high-level (T3) SCI, while the immune organs of rats with low-level SCI showed gradual recovery. T3-SCI was accompanied by a decrease in NE expression and a significant increase in GC levels.

"Omics" technology may be the main means of target identification and screening, and unbiased analysis of transcriptomics can be used as an objective exploration of disease targets. A number of studies have focused on the use of multiomics [31] and spatiotemporal expression of local tissues of SCI [32–35], and previous studies have explored transcriptional, protein and metabolic changes in local injury and carried out in-depth research on understanding the pathological changes, molecular mechanisms and spinal cord repair of local injuries [31, 36, 37]. Previous studies have shown that day 7 is the subacute phase of SCI, which is a critical period for secondary injury to SCI and an important time point for treatment [36, 38]. A recent study based on the single-cell transcriptome of SCI mice suggests that day 7 of injury is the peak of microglia, neuroinflammation, and immune response [39]. First, this study performed bulk transcriptome analysis on day 7 after SCI to observe systemic immune changes. Second, SCI-IDS may be attributed to loss of inhibitory control of the spinal cord after injury, leading to neuroendocrine disorders. Therefore, to understand the neuroendocrine and immune changes following SCI, for the first time, we analysed the molecular profiles of the hypothalamus and adrenal glands in the HPA axis, as well as the genetic changes in the immune organ spleen, by unbiased transcriptome analysis to reveal the possible mechanisms behind immune deficiency after injury, with tissue spatial specificity. It is well known that SCI-IDS is typically injury level dependent [6], and here, we selected high-level (Th3) and low-level (Th10) rat models of complete thoracic SCI to reveal the neurogenic mechanism of SCI-IDS, as the adrenal glands almost lose control of the supraspinal center after high levels of SCI. Compared with stroke and traumatic brain injury, the spinal cord is relatively structurally unified as a liaison station between the supraspinal center and the periphery, so SCI provides a favorable neuroanatomical method to decipher the neuroendocrine mechanisms of immune-related organs.

Unbiased transcriptome analysis and histopathological staining of multiple tissues (including the hypothalamus, spinal cord, adrenal glands and spleen) after injury to different spinal cord levels revealed neuroendocrine-mediated immunodeficiency after high-level SCI. T3-SCI induced uncontrolled and progressive inflammation in the CNS (the hippocampus, hypothalamus, and spinal cord) and persistent impairment of the immune function of peripheral organs (the spleen and adrenal glands). Moreover, T3-SCI inhibited neurotransmission via Gi-GPCRs and neuropeptide production in the CNS. Finally, T3 injury impaired adrenal gland-mediated circadian regulation and cortisol secretion. In conclusion, this study identified the main molecular changes after injury to different spinal cord segments based on unbiased transcriptome analysis and preliminarily delineated the differentially regulated neuroendocrine–immune network associated with SCI-IDS.

### The contradiction between neuroinflammation in the CNS and systemic immunosuppression after high-level SCI

SCI triggers a systemic inflammatory response, which is particularly strong in the spinal canal and widespread in the spinal cord [25, 40]. Studies have shown that inflammation within the spinal canal persists indefinitely and that autoimmune pathology depends on the extent of injury [41]. We previously found that activation of an inflammatory cascade secondary to spinal cord injury resulted in significant white matter loss at the distal end of the spinal cord [25]. In this study, transcriptome analysis showed that CNS neuroinflammation was more severe after high-level SCI than after low-level SCI. Microglia are present in all CNS tissues associated with the HPA axis and secrete factors that synergistically stimulate HPA axis activity in an autocrine manner. Studies have determined that activated microglia disrupt BBB function by releasing molecules that increase permeability and induce inflammation [42]. Injury-induced activation of microglia/macrophages disrupts the blood‒spinal cord barrier, hinders the restoration of key mechanisms of CNS immune privilege, further exacerbates inflammatory infiltration in the upper spinal cord, and affects brain neural networks. In particular, complex neural networks receive and integrate information transmitted from hypothalamic regions via various peripheral hormones, including those in the HPA axis and the autonomic nervous system (ANS). Here, an inflammatory response of the same degree was observed in the hippocampus as in the hypothalamus, and studies suggest that the hippocampus makes direct and indirect polysynaptic connections with the PVN; moreover, the hippocampus has been identified as a particularly important region for activating the ANS and HPA. Furthermore, activated astrocytes interact with neurons and microglia and act as sensors of immune information and components of tripartite synapses to integrate immune and neural signals [43]. Finally, presynaptic Gi-coupled GPCRs and neuropeptides (including opioid receptors) reduce neurotransmitter release by targeting complex mechanisms of ion channels and vesicles in a synaptic- and activity-dependent manner, ultimately reducing the expression of PSD-95 via neurotransmission.

Nevertheless, immunosuppression therapy for SCI has been largely unsuccessful due to the limited concentration of neuroinflammation in limited intraspinal "compartmentalization" studies [44]. In modern view, the inflammatory response caused by SCI acts as a double-edged sword because inflammation and immunity exert both beneficial (plasticity-enhancing) and harmful (glial cell degeneration and neurodegeneration; secondary injury) effects, and these functions change over time [45, 46]. Intriguingly, inflammation not only leads to the spread of damage throughout the spinal cord but is also an important cause of multiorgan dysfunction, especially organ atrophy, which mediates immunodeficiency and hematopoietic dysfunction [47]. The structure and function of the CNS are affected by systemic immune challenges, so the dynamic interaction between peripheral immune deficiency and intraspinal inflammation must be revisited to maximize immune homeostasis recovery after SCI.

In a clinical cohort study, RNA-seq of blood samples from patients with SCI showed significant downregulation of genes associated with natural killer (NK) cells and upregulation of genes related to proinflammatory Toll-like receptor signalling pathways [41]. In this work, high-level SCI results in complete inhibition of the immune function of peripheral endocrine-immune organs (the spleen and adrenal glands); the injury inhibits the adaptive immune responses of the spleen and adrenal glands, including immune organ atrophy, B-cell development and responses, antibody production, and MHC-II-mediated immunity. MHC-II is typically considered a marker of granulocyte and monocyte activation, which subsequently promotes T-cell activation [28, 48]. Locally, in the adrenal gland, high expression of GCs after high-level SCI is related to increased adrenal-mediated regulation of circadian rhythm, and the autocrine effect of high expression of GCs on immune cells is related to cell survival [49]. For example, GCs can regulate MHC-II expression and were previously shown to reduce MHC-II gene expression on monocytes; reticular band cells of the adrenal cortex express MHC/HLA class II molecules, and leukocytes interact directly with the adrenal glands [50]. GCs also indirectly negatively affect the B-cell response by reducing the number of helper T cells and inhibiting their activation while also inhibiting the function of antigen-presenting dendritic cells [52]. Via the brain-spleen immune axis, the PVN can control GC signalling to force lymphocytes and monocytes to migrate into the bone marrow, reducing their number and maturation [51]. Re-establishing near-baseline GC levels after SCI can prevent pneumonia due to SCI-IDS [10]. However, studies suggest that targeting stress-related axes (the HPA axis, SAM axis, and neurotrophic factor/neuropeptide axis) has no beneficial effect on or is even detrimental to functional recovery in mice after SCI [10, 53]. Therefore, restoring basal levels of endogenous GC release may be an effective therapeutic strategy for SCI-IDS.

### Mechanism underlying the disruption of the connection between the hypothalamus and the adrenal glands in the HPA axis after high-level SCI

Normal connections between the CNS and the immune system are disrupted after SCI, resulting in SCI-associated immunosuppressive syndrome, which enhances susceptibility to infection depending on the level of the lesion. Loss of control of downstream immune organs (the spleen and adrenal glands) by the upper spinal cord is largely due to disruption of neuronal afferents after injury [54–58]. Studies have shown that this enhanced susceptibility to infection is mediated by a central mechanism [7, 8, 58–60]. Many scientists have tried to elucidate the mechanism underlying neuroendocrine-immune crosstalk, and it is still unclear. This study preliminarily reveals that after high-level SCI, splenic immune function is impaired, adrenal-mediated cortisol production is increased, the production of the hormone ACTH upstream of the HPA axis is negatively regulated, the expression of transmitters such as GPCRs and neuropeptides are decreased, and the inflammatory response is enhanced. Studies have shown that primary hypercortisolism, which is not caused by ACTH, occurs following SCI and that ACTH expression tends to be suppressed after high-level SCI [10, 61]. We analysed the causes of these changes. The progressive downregulation of neuropeptide transmitters, such as NPY and the endogenous opioid receptor OPRM1, reduces the central synthesis of ACTH and causes HPA axis disruption. Moreover, after SCI, the homeostatic function of the adrenal glands is impaired, abolishing their ability to regulate the hypothalamus, and hypercortisolemia is caused by adrenal denervation (CNS decompression), resulting in inhibition of ACTH expression through a negative feedback mechanism and eventually to a high degree of adrenal autonomy.

### Adrenal gland-mediated circadian rhythm disturbances after high-level SCI further exacerbate immunodeficiency

Normally, initiation of the stress response causes damage to the body, and glucocorticoids inhibit the function and migration of white blood cells, thereby reducing the production of inflammatory mediators and cytokines and reducing their effects on the body. However, excessive suppression leads to immunodeficiency. The balance between immunodeficiency and immunopathology is achieved through a combination of endogenous anti-inflammatory and proinflammatory mechanisms [28, 62].

Here, we observed the phenomenon of overreaction of central congenital inflammation with total suppression of peripheral adaptive immunity. Through adrenal transcriptome analysis, we observed a simultaneous increase in circadian rhythm regulation and cortisol levels after high-level SCI accompanied by MHC-II-related immunodeficiency, suggesting that circadian rhythm disturbance aggravates immune deficiency. Studies have shown that sympathetic innervation of the adrenal cortex controls the circadian sensitivity of cortical cells to ACTH, which in turn regulates the circadian rhythm and GC release [63]. However, disruption of preganglionic neurons contributes to loss of inhibition of circadian rhythm regulation. Therefore, circadian rhythm disturbances of the adrenal glands after high levels of SCI have the potential to further exacerbate immune deficiencies.

### The Tai Chi mechanism underlying the complex regulatory effect of the neuroendocrine system on SCI-IDS

The mechanism by which SCI causes immune deficiency is complex and involves dysregulation of multiple systems and organs. Imbalance of the neuroendocrine immunomodulatory axis (NIA) in any direction can lead to immune dysfunction [64]. The NIA is a robust system that is mutually regulated and highly coordinated, and it maintains homeostasis and ensures normal physiological functions, similar to the traditional theory of Chinese Tai Chi. Although it is always changing, it always pursues balance and gives rhythm. This study uses multitissue transcriptome analysis to preliminarily analyse differences in gene expression between the hypothalamus, spinal cord, adrenal glands and spleen after SCI, revealing the presence of a regulatory network of neuroendocrine immunity after high-level SCI rather than compartmentalization of damage.

**Fig. 10:**
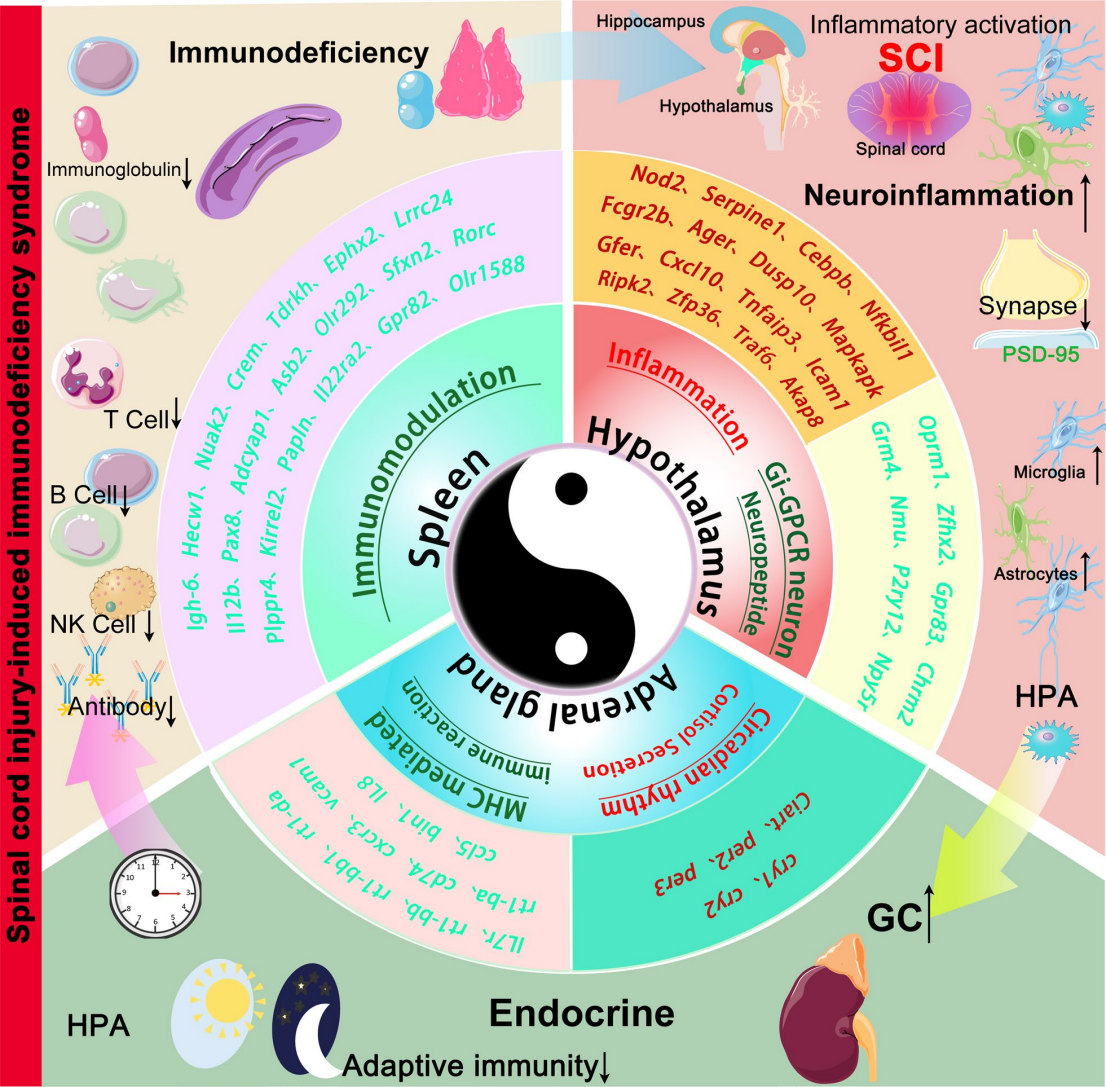
The Tai Chi mechanism underlying the complex regulatory effect of the neuroendocrine system on SCI-IDS. The Tai Chi diagram shows that the main tissues and organs involved in neuroendocrine immunity are the hypothalamus, adrenal glands and spleen, which are also important components of the HPA axis (from the inside out, the most central). Multitissue transcriptome analysis showed that the pathway-gene target map was composed of DEGs and the GO pathways in which they were enriched after T3-SCI; red indicates upregulation, and green indicates downregulation (second and third layers). Possible mechanisms of NIA regulation after SCI (outermost)

The observed neuroinflammation in the CNS and systemic immunosuppression after high-level spinal cord injury, as shown in Fig. 10, are contradictory. The right panel shows uncontrolled neuroinflammation in the CNS, and the left panel shows impairment of the adaptive immune function of peripheral organs. Moreover, the SCI-mediated disruption of the connection between the hypothalamus and the adrenal glands in the HPA axis, hypercortisolemia and the regulatory uncoupling of the hypothalamus may be due to the loss of control of neuronal Gi-GPCRs and neuropeptides. Finally, injury induces circadian rhythm disturbances in the adrenal glands, further exacerbating immunodeficiency. The exact mechanism then needs to be regulated and validated by pharmacological, optogenetic, or chemogenetic regulatory targets.

## Conclusion

Remodelling of immune homeostasis after SCI is the basis of nerve repair and functional recovery. Neuroinflammation caused by SCI persists indefinitely, hindering nerve repair; persistent system-wide immunosuppression in the periphery results in increased susceptibility to infection, leading to poor neurological prognosis. In this study, unbiased transcriptomic analysis initially revealed and validated a gene network responsible for neuroendocrine immunomodulation after high-level SCI.

Through analysis of multiple tissues via various techniques and verification, a mechanism of SCI-IDS is proposed; specifically, the spread of neuroinflammation to the CNS after injury inhibits neurotransmission through Gi-GPCRs in the HPA axis and neuropeptide production in the hypothalamus. This further leads to autonomous regulation of the dissociated adrenal glands due to disruption of the connection between the hypothalamus and the adrenal glands and the disturbance of circadian rhythm and finally leads to hypercortisolemia, causing complete suppression of peripheral adaptive immunity.

### Supplementary Information

**Additional file 1: **Summary of Illumina's postsequencing sequence assembly.

**Additional file 2:** The principal component analysis between different samples.

**Additional file 3: **The GO terms enriched of hypothalamus in 352 upregulated DEGs.

**Additional file 4: **The GO terms enriched of hypothalamus in 185 downregulated DEGs.

**Additional file 5: **The GO terms enriched of spinal cord in 741upregulated DEGs.

**Additional file 6: **The GO terms enriched of spinal cord in 681downregulated DEGs.

**Additional file 7: **The GO terms enriched of adrenal gland in 248 upregulated DEGs.

**Additional file 8: **The GO terms enriched of adrenal gland in 222 downregulated DEGs.

## Data Availability

(1) RNA-Seq raw data have been deposited in the NCBI Sequence Read Archive (SRA, https://submit.ncbi.nlm.nih.gov/subs/sra/SUB13039409/overview). Accession IDs for *Rattus norvegicus* BioProject = PRJNA953749; BioSample = SAMN34122650-SAMN 34122675 (13 objects of adrenal gland) (13 objects). (2) RNA-Seq raw data have been deposited in the NCBI Sequence Read Archive (SRA, https://submit.ncbi.nlm.nih.gov/subs/sra/SUB13054007/overview). Accession IDs for Rattus norvegicus BioProject = PRJNA953752; BioSample = SAMN34122695-SAMN34122720 (13 objects of hypothalamus). (3) RNA-Seq raw data have been deposited in the NCBI Sequence Read Archive (SRA, https://submit.ncbi.nlm.nih.gov/subs/sra/SUB13054026/overview). Accession IDs for *Rattus norvegicus* BioProject = PRJNA953782; BioSample = 34123816-SAMN34123839 (13 objects of spleen). (4) RNA-Seq raw data have been deposited in the NCBI Sequence Read Archive (SRA, https://submit.ncbi.nlm.nih.gov/subs/sra/SUB5917896/overview). Accession IDs for *Rattus norvegicus* BioProject = PRJNA552942; BioSample = SAMN12222084-SAMN12222089 (6 objects of spinal cord).

## Abbreviations

SCI: Spinal cord injury
RNA-seq: RNA sequencing
SCI-IDS: Spinal cord injury-induced immunodeficiency syndrome
HPA: Hypothalamo–pituitary–adrenal axis
DEGs: Differentially expressed genes
GO: Gene Ontology
CNS: Central nervous system
BBB: Blood brain barrier
CVO: Circumventricular organs
TH: Tyrosine hydroxylase
GC: Glucocorticoid
CA: Catecholamine
CIDS: Central nervous system injury-induced immunodeficiency syndrome
SBP: Systolic blood pressure
IOD: Integrated optical density
NE: Norepinephrine
ACTH: Adrenocorticotropic hormone
TPM: Transcripts perkilobase million
PCA: Principal component analysis
RP: Red pulp
WP: White pulp
PVN: Paraventricular nucleus
DG: Dentate gyrus
IBA1: Ionized calcium binding adapter molecule 1
GFAP: Glial fibrillary acidic protein
PSD-95: Postsynaptic density 95
DC: Degree centrality
Zr: Zona reticularis
Zf: Zona fasciculata
Zg: Zona glomerulosa
Gi-GPCR: Gi mediated G protein coupled receptor
ANS: Autonomic nervous system
MHC: Major histocompatibility complex
NIA: Neuroendocrine immunomodulatory axis
