# Supplementary figures and images for: Unbiased multitissue transcriptomic analysis reveals complex neuroendocrine regulatory networks mediated by spinal cord injury-induced immunodeficiency
Source: J Neuroinflammation. 2023 Sep 30;20:219. doi: 10.1186/s12974-023-02906-7 (PMC10543323; doi:10.1186/s12974-023-02906-7)

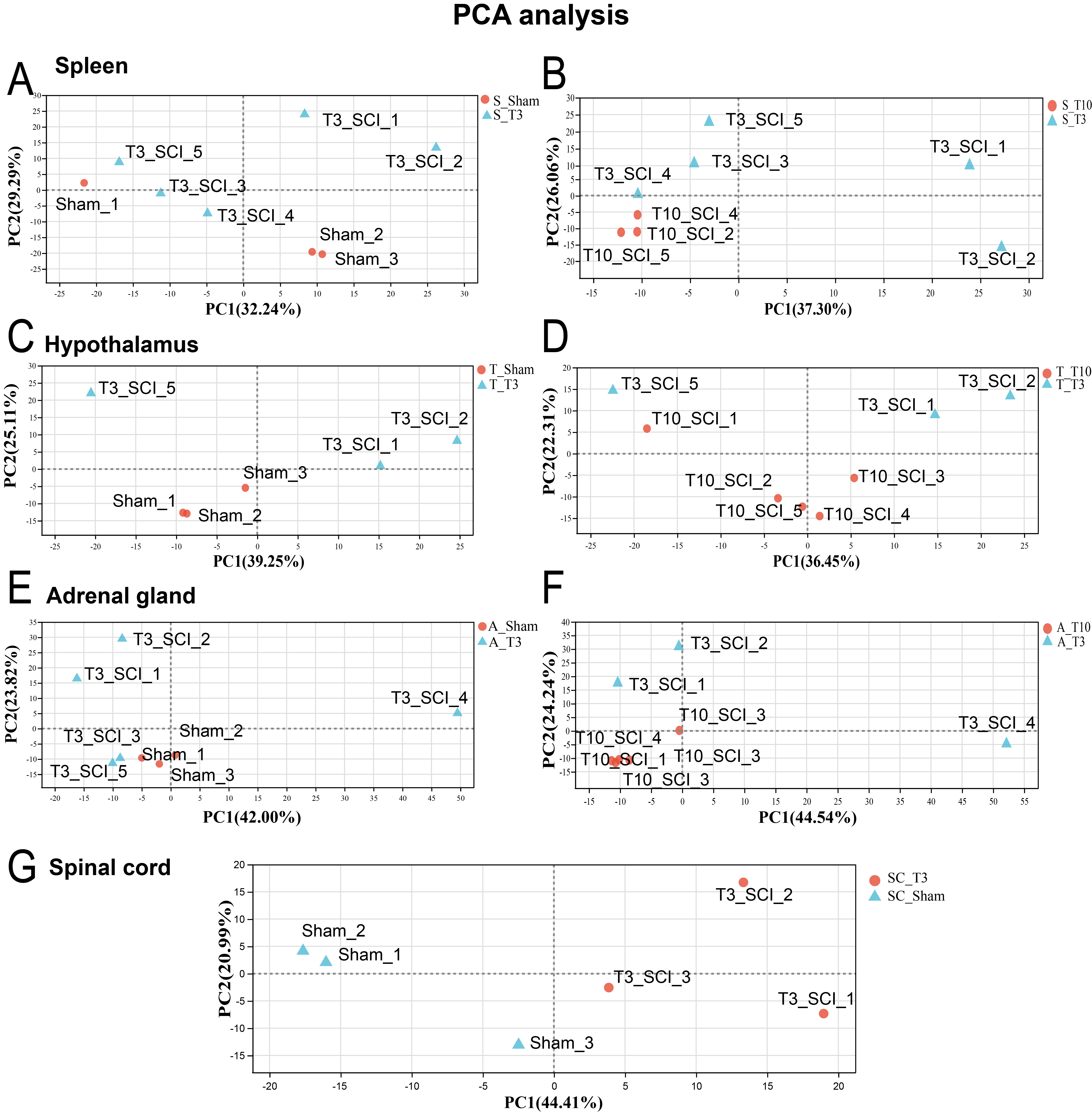

Supplement: Supplementary file 2 — Additional file 2: The principal component analysis between different samples. [file 12974_2023_2906_MOESM2_ESM.tif]
